# Circadian rhythm regulates the function of immune cells and participates in the development of tumors

**DOI:** 10.1038/s41420-024-01960-1

**Published:** 2024-04-27

**Authors:** Yuen Zeng, Zichan Guo, Mengqi Wu, Fulin Chen, Lihua Chen

**Affiliations:** 1 Department of Immunology, School of Basic Medical Sciences, Air Force Medical University, Xi’an, China; 2 Faculty of Life Sciences, Northwest University, Xi’an, China

**Keywords:** Immunoediting, Circadian rhythms

## Abstract

Circadian rhythms are present in almost all cells and play a crucial role in regulating various biological processes. Maintaining a stable circadian rhythm is essential for overall health. Disruption of this rhythm can alter the expression of clock genes and cancer-related genes, and affect many metabolic pathways and factors, thereby affecting the function of the immune system and contributing to the occurrence and progression of tumors. This paper aims to elucidate the regulatory effects of BMAL1, clock and other clock genes on immune cells, and reveal the molecular mechanism of circadian rhythm’s involvement in tumor and its microenvironment regulation. A deeper understanding of circadian rhythms has the potential to provide new strategies for the treatment of cancer and other immune-related diseases.

## Facts

- Circadian rhythms profoundly affect various physiological processes in the body.
- Circadian rhythms influence the maturation, localization, and function of immune cells in a variety of ways (including genes, adhesion molecules, cytokines, etc.).
- The immune system can respond by influencing circadian rhythms.
- Circadian rhythms are involved in cell cycle regulation.
- The components related to circadian rhythm in tumor cells and their microenvironment are altered, and there is an epidemiological link between cancer risk and circadian rhythm gene expression.

## Open questions

- Can circadian rhythm considerations be used to optimize clinical administration, nutrition, and surgical timing to optimize patient outcomes?
- Can circadian rhythms be integrated into drug use and surgical timing for better outcomes?
- Can we predict cancer risk by detecting the expression status of circadian rhythm genes in combination with other markers?

## Introduction

The 24-h cycle generated by the earth’s rotation has caused the evolution of circadian rhythms of almost all life forms on the earth [1]. This evolutionarily conservative time maintenance mechanism enables organisms to adapt to the ever-changing environment [2, 3]. In essence, the circadian clock constitutes an automatic regulatory sequence for the expression, accumulation and degradation of clock gene products, forming an autonomous molecular oscillator [3]. The molecular clock in the animals controls the expression of genes that are output throughout the body, thereby controlling the activity and function of different cells and organs over time [4]. Therefore, mutations in the clock gene can lead to changes in various biological rhythmic behaviors [5].

Almost all physiological activities of mammals are regulated by the body’s biological clock, including sleep-wake, eating-fasting, and activity-rest cycle [2]. Its master clock is located in the suprachiasmatic nucleus (SCN) of the hypothalamus, which can process light input signals and transmit timing information to other parts of the body [1]. Peripheral clocks are located in other tissues and organs, some are strictly dependent on the regulation of the central clock (such as the liver), and some remain relatively independent [6]. SCN neurons maintain synchronization and are coupled to each other through synaptic connections [7]. Synchronization signals are transmitted from the SCN clock to the peripheral clock through neural and hormonal stimulation to achieve a coherent rhythm throughout the organism [8, 9].

In humans, the physiological and behavioral circadian rhythms have evolved to form the biological clock that supports the occurrence of wakefulness during the daytime and promotes sleep during the night. Various aspects such as body temperature, metabolism, immune response, etc., exhibit circadian rhythms, and sleep is also a rhythmic behavior. Therefore, disruption of the circadian rhythm can directly impact sleep, and research has shown that light exposure can directly promote wakefulness and reduce sleep propensity [10]. Disturbances in the circadian rhythm can lead to various negative consequences, one of which is Circadian Rhythm Sleep-Wake Disorders (CRSWD). CRSWD is a distinct category of sleep disorders (sleep disorders mainly include insomnia, circadian rhythm disorders, sleep-disordered breathing, hypersomnia/narcolepsy, parasomnias, and restless legs syndrome/periodic limb movement disorder [11]). According to the classification criteria of ICSD-3, CRSWDs can generally be divided into two categories: one resulting from changes in the endogenous circadian rhythm system and the other due to a mismatch between sleep-wake time and internal circadian rhythm caused by environmental changes [12]. Melatonin, as an endogenous circadian factor that promotes sleep, when used at the appropriate time for treatment, can regulate the sleep-wake cycle [13]. In a placebo-controlled double-blind crossover trial involving 30 patients, it was observed that when patients took 5 mg of melatonin, their endogenous melatonin secretion occurred 1.5 h earlier. PSG analysis indicated that these patients showed advances and shortened sleep onset latency during the treatment period, without significant changes in sleep architecture. Furthermore, compared to before treatment, patients exhibited increased alertness upon waking in the morning and significant improvements in quality of life [14].

The core clock of mammalian circadian rhythms consists of a group of conservative transcription factors, in which the core is the key regulatory transcription activator circadian locomotor output cycles protein kaput (CLOCK), brain and muscle ARNT-like 1 (BMAL1) and neuronal PAS domain-containing protein 2 (neuronal PAS2) [1]. They form heterodimer complex CLOCK-BMAL1 and neuron PAS2-BMAL2 and they act on E-box element on target genes [1]. These target genes include circadian rhythm transcription factors period circadian protein homologue 1 (PER1) and PER2, cryptochrome 1 (CRY1) and CRY2, REV-ERBα (NR1D1) and REV-ERBβ (NR1D2), etc., all of which act as an inhibitor to the core clock [1].

Transcription of *Per* and *Cry* genes is induced by the CLOCK/BMAL1 complex. After their proteins being synthesized, they accumulate in the cytoplasm and form heterodimers that shuttle between the nucleus and cytoplasm [15]. When the level of PER/CRY complexes in the nucleus is sufficiently high, they inhibit the transcription of E-box genes (including their own genes) via blocking CLOCK/BMAL1-mediated transcription [3]. Gene mutations that change the phosphorylation state, mobility, or degradation of PER and CRY proteins may affect the clock speed [16, 17]. PER proteins are subjected to successive phosphorylation events of multiple residues by CK1δ and CK1ε and by CK2 [17–25], leading to the change of their susceptibility to proteasomal degradation mediated by E3 ubiquitin ligase β-TrCP [26, 27]. Whether phosphorylation promotes protein stability or degradation depends not only on the modified residues, but also on the subtle differences in the kinases responsible [3]. For example, phosphorylation of CK1δ selective splicing variants has an inverse effect on the stability of PER2 and the length of the circadian rhythm cycle [28]. In addition, the length of the circadian rhythm cycle is closely related to CRY protein abundance [29–36]. Phosphorylated CRY1 and CRY2 become targets for E3 ubiquitin ligases FBXL3 and FBXL21, while JMJD5 can promote CRY1–FBXL3 complexes to target proteasomes, thereby regulating the length of the circadian rhythm cycle via affecting the degradation of CRY proteins [37–42].

**Fig. 1:**
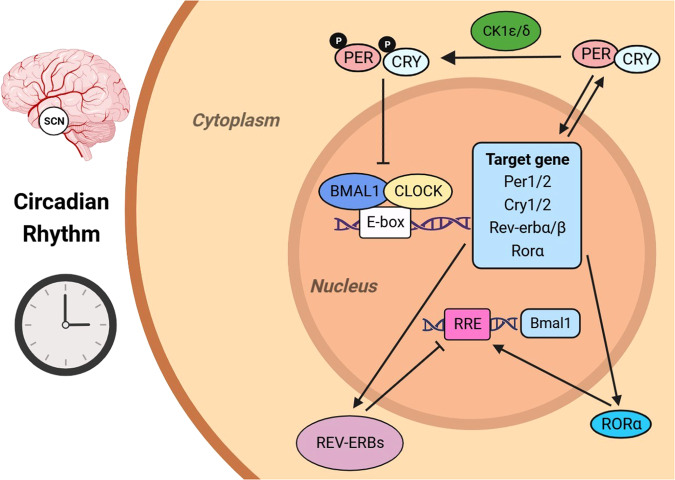
Circadian rhythm mechanism. The master clock affecting the body’s circadian rhythm is located in the hypothalamic suprachiasmatic nucleus (SCN), and the synchronization signal is transmitted from the SCN clock to the peripheral clock. The heterodimer complex CLOCK/BMAL1 acts on E-box elements on target genes. After synthesis, PER and CRY accumulate in the cytoplasm and form heterodimers that shuttle between the nucleus and cytoplasm. After phosphorylation under the action of CK1δ and CK1ε, they inhibit the transcription of E-box genes by blocking CLOCK/BMAL1-mediated transcription; REV-ERBα inhibits the expression of *Bmal1* by binding to the ROR responsive element (RRE) in the promoter, while RORα has the opposite activation effect.

Nuclear receptors REV-ERBα and REV-ERBβ are not only key components of the molecular circadian clock, but also major regulators of metabolism and mood [43–45]. The retinoid-related orphan receptor (ROR) nuclear receptor drives the expression of BMAL1 in the feedforward loop [1]. REV-ERBα inhibits *Bmal1* gene expression by binding to the ROR response elements (RRE) located in these gene promoters, and to a lesser extent inhibits *Clock* gene expression [46–49]. RORα shares DNA binding sites with REV-ERBs [1], and when REV-ERBα inhibits the transcription of *Bmal1*, the RORα activates it [50]. Pharmacological activation of RORα and RORγ, which are the antagonists of REV-ERBα and REV-ERBβ in the molecular clock, can enhance rhythmic oscillations [51, 52], thereby demonstrating therapeutic potential in mouse models of Alzheimer’s disease, Parkinson’s disease, depression, and obesity [51, 53–55] (Fig. 1).

Circadian rhythms are involved in regulating almost every kind of physiological activity, including mood regulation, cell cycle control, metabolism, and immune responses. In some pathological conditions, the expression of clock genes also changed. Disruption of circadian rhythm may lead to abnormal cell proliferation, increased gene mutation, and resistance to apoptosis [56, 57], thus participating in the occurrence and development of a variety of diseases, including metabolic, cardiovascular, psychiatric diseases, and cancer. This paper systematically summarizes the molecular mechanism of circadian rhythm. The effects of relevant molecules on the immune system and cancer development; it will help open up new ideas for the treatment of certain diseases from the perspective of circadian rhythm.

## Effect of circadian rhythms on immune cells

### Macrophages

Macrophages are one of the important innate immune cells, which induce a series of signal cascade reactions through Toll-like receptors (TLRs) on the cell surface to convert cells into immune active state [58]. A study found that the death caused by *E. coli* endotoxin changed with the injection time, indicating that the TLR4 signal was under the control of circadian rhythms [59]. In addition, in the TLR9-dependent septicemia model, stronger lethality was observed on the day when TLR9 expression was highest in spleen macrophages and B cells [60]. The expression of *Tlr2* and *Tlr6* mRNA in spleen macrophages also showed rhythm [60]. Therefore, the circadian rhythms of TLR and its downstream signal pathways may be one of the important mechanisms guiding circadian inflammation.

Macrophages can polarize into pro-inflammatory M1 phenotype with lipopolysaccharide (LPS) or anti-inflammatory M2 phenotype with IL-4, altering glycolysis and oxidative phosphorylation [61, 62]. BMAL1 inhibits septicemia development by affecting glycolysis metabolism. Loss of BMAL1 in macrophages reduces glycolysis control and increases infective shock mediated by PD-L1/T cells [63]. Macrophages lacking clock genes Per1 and Per2 exhibit a pro-inflammatory phenotype similar to M1 [64], while the hormone melatonin promotes M2 phenotype production via RORα and metabolite signals [65, 66]. Proteins Nfil3 and Dbp from circadian rhythm genes competitively bind to the Il12b promoter, inhibiting and enhancing expression, respectively [63, 67]. The oscillation of these circadian rhythm proteins provides a rhythmic response to LPS [63].

Macrophages secrete cytokines and chemokines to participate in inflammation. REV-ERBα inhibits macrophage inflammation through various mechanisms [63]. The lack of REV-ERBα increases inflammation in alveolar macrophages [68], while REV-ERBα agonists inhibit inflammatory gene expression in macrophages [69]. REV-ERBα also regulates inflammatory markers in microglia [70] and colitis models [70, 71]. It directly regulates Nlrp3 mRNA in a hepatitis model [72]. The PER/CRY complex is another mechanism for inhibiting inflammatory mediators in macrophages. Deletion of Per1 increases Ccr2 expression and migration ability [73], while deletion of Per2 increases TNFα and IL-12 production [74]. CRY negatively regulates the cAMP-PKA-NF-κB pathway [75]. Its deficiency leads to upregulation of Il6, Tnfα, and inos. Studies targeting REV-ERBα found that GSK4112 inhibited NLRP3 expression and IL-1β production in an LPS-induced inflammation model [76]. Disruption of circadian rhythm and KLF4 downregulation affect macrophage function in aging mice [77], highlighting the regulatory role of circadian rhythm.

Circadian rhythm can affect the activation of toll-like receptor (TLRs) signaling pathway on the surface of macrophages, regulate the immune activity and inflammatory response of macrophages, and also affect the polarization state and metabolic pathway selection of macrophages, thereby regulating their ability to participate in inflammatory response and immune function. This interaction may be one of the important mechanisms guiding circadian-related inflammation.

### Dendritic cells

Dendritic cells (DCs) are antigen-presenting cells whose activity is influenced by circadian rhythm. In Rev-erbα KO or Rev-erbβ KO mouse bone marrow, DCs extracted show increased expression of MHCII and CD86, indicating maturity [78]. Conversely, the presence of REV-ERB agonist leads to the opposite effect. Defective migration of Bmal1 KO DCs into the spleen was observed, resulting in reduced CD8 T cell activation [79]. In addition, DCs exhibit stronger migration from skin to lymphatic vessels during the day compared to nighttime, and the deletion of Bmal1 gene eliminates this rhythmic migration pattern [80].

DCs also play a vital role in anti-tumor immunity. RNA sequencing analysis reveals that clock genes and clock control genes are expressed rhythmically in CD11c^+^MHCII^hi^ migrating DC subsets collected after tumor transplantation or under sham conditions [81]. The rhythmic production of DC costimulatory factors may contribute to the rhythmic activation of CD8^+^ T cells. The expression of CD80 in different CD11c^+^ subgroups is time-dependent and controlled by the circadian clock mechanism. Deletion of BMAL1 eliminates the time difference in CD80 expression [81]. The proliferation of OT-I CD8^+^ T cells is dependent on the rhythmic phase of DCs. BMDCs with BMAL1 deficiency fail to induce rhythmic proliferation of OT-I CD8^+^ T cells. Treatment with anti-CD80 antibody eliminates the time difference in OT-I CD8^+^ T cell proliferation, confirming the correlation between CD80 and the rhythmic response of CD8^+^ T cells [81]. Chromatin Immunoprecipitation (ChIP) confirms the rhythmic binding of BMAL1 to the promoter region of the CD80 gene, indicating that CD80 is directly controlled by the clock gene BMAL1 [81].

Circadian rhythms influence the activity and function of dendritic cells (DCs), including maturity, antigen presentation, and migration. Circadian mechanisms also regulate the role of DCs in anti-tumor immunity, including the rhythmic expression of clock genes and related factors and the production of factors associated with the activation of CD8^+^ T cells. CD80 plays an important role in the circadian rhythm controlled CD8^+^ T cell response and is directly transcriptionally controlled by the clock.

### B lymphocytes

The relationship between biological clock genes and lymphocyte development has been well established. Deficiency of BMAL1 negatively affects B cell development, leading to a decrease in their number in the blood and spleen of animals with knocked out BMAL1 gene [82]. However, adoptive transfer experiments have shown that it is not the internal clock of B cells but the loss of Bmal1 in the bone marrow microenvironment that hinders B cell differentiation [82]. On the other hand, mice with B-cell-specific Bmal1 deficiency do not exhibit any changes in B cell differentiation or function [83], suggesting that the influence of clock genes on B cell development is dependent on the microenvironment.

Some research has found that lymphocytes, including B cells, in mouse lymph nodes exhibit circadian rhythm oscillation. The highest number of lymphocytes is observed at the beginning of the active period (nighttime for mice). This rhythmic oscillation is due to the rhythmic homing of cells from blood into lymph nodes and their rhythmic egress from lymph nodes to efferent lymph vessels [84–86]. C-C-chemokine receptor 7 (CCR7) is a key regulatory factor that shows diurnal oscillation in both T cells and B cells [84]. In addition, the migration of B cells to lymph nodes also relies on other receptors, such as C-X-C-chemokine receptor 4 (CXCR4) and CXCR5 [87, 88]. The expression levels of CCR7 and CCL21 peak before and after the onset of nocturnal disease, leading to the recruitment of B cells. This process involves the microenvironment and lymphocytes [84]. Sphingosine 1-phosphate receptor 1 (S1P1) plays a regulatory role in the rhythmic export of cells to efferent lymph vessels [84]. Its rhythmic expression promotes the egress of lymphocytes from lymph nodes in response to the chemotactic lipid sphingosine 1-phosphate [89].

Circadian rhythm disorders from irregular work and rest can damage B cell function, affecting the immune system. A study discovered that day-night shift rotation (DNSR) reduces nurses’ IL-10 B cell (B10 cell) immunosuppressive ability and impairs Tr1 production in B10 cells [90]. Following DNSR, although B10 cell numbers remain unchanged, IL-10 levels decrease. RNA sequencing reveals CLOCK as one of B10 cells’ most highly expressed genes post-DNSR. CLOCK levels correlate negatively with IL-10 mRNA levels. In addition, bB10 cells (pre-DNSR B10 cells) inhibit CD4^+^ T cell proliferation and induce Tr1 cells, but this immunoregulatory ability weakens after DNSR [90].

At present, the research on the connection between circadian rhythm and B lymphocytes is still little, and the regulation and influence of circadian rhythm on B cells need to be further explored.

### T lymphocytes

#### CD8^+^ T cells

CD8^+^ T cells are one of the important cells that participate in adaptive immune response, and they eliminate intracellular infection, control chronic infection, and eliminate tumor by producing cytokines (such as IL-2, IFN-γ, and TNF-α) and cytotoxic molecules (perforin and granzyme) [91]. A study confirmed the expression of *Per1* in CD8^+^ T cells of mouse spleen and the continuous rhythmic expression of PER2 protein in vitro-cultured CD8^+^ T cells [79, 83], indicating that CD8^+^ T cells have endogenous and cellular autonomic circadian clock.

T cells exhibit rhythmic expression of chemokine receptors and surface molecules, leading to migratory patterns and daily oscillations in organs. Cortisol regulates CXCR4 expression on T cells, impacting migration to CXCL2-expressing sites [92, 93], thus regulating Naive T cell levels in human blood. Naive T cells peak at night and are low during the day. Human effector CD8^+^ T cells peak during the day under catecholamine control [93]. In mice, CD4^+^ and CD8^+^ T cells are highest at the start of the day (ZT4-5) and lowest at the start of the night (ZT16) [94, 95]. Lymph nodes show an opposite rhythm, indicating T cell redistribution from blood to lymph nodes at night [84, 86]. This rhythmic migration is due to rhythmic CCR7 expression in CD8^+^ T cells and CCL21 (CCR7 ligand) in lymph nodes [91]. In addition, CD127 expression in CD8^+^ T cells induces CXCL4 (CXCL12 receptor) expression via IL-7, mediating daily oscillation in lymph organs [86].

Circadian rhythm mainly influences T cell proliferation. Stimulation of CD8^+^ T cells collected from mouse lymph nodes during a 24-h dark/dark cycle at night results in faster proliferation [96]. However, the proliferation rhythm is eliminated in T cells from mice with negative transcription factor CLOCK and deletion of the essential clock gene Bmal1 in mature CD8^+^T cells [79, 96]. A study using high-throughput RNA sequencing data from the Cancer Genome Atlas discovered that circadian rhythm disruption in tumors is associated with increased tumor-associated T cell dysfunction in human cancers. The study analyzed 716 samples from 29 different types of cancer. In most cancer types, the expression of a negative regulator of the circadian clock was downregulated, while a positive regulator, ARNTL2, was upregulated. This indicates that tumors lose the oscillating mRNA expression of the circadian clock gene. In addition, high expression of the clock gene correlated positively with levels of T cell dysfunction markers such as PD-1 and CTLA-4 in tumor samples. These findings suggest a connection between dysregulated clock gene expression and T cell dysfunction in aging and cancer, implying that circadian rhythms influence adaptive immune cell aging [97].

Disruption of circadian rhythms in tumors may lead to impaired function of tumor-associated T cells. In addition, T cells exhibit different chemotaxis and migration patterns under circadian rhythms, and the rhythmic production of cortisol and catechol amines regulates the migration and localization of T cells. The proliferation of T cells is also influenced by circadian rhythms, showing a faster rate of proliferation at night. These research findings suggest that circadian rhythms have important effects on the activity, migration, and proliferation of T cells, and disruption of circadian rhythms may impact immune responses, including tumor immune responses.

#### Helper T cells

Some studies have found that the specific circadian rhythm gene, rather than the main circadian rhythm regulator (BMAL1/CLOCK), regulates the specific CD4 Tn response [98]. In CD4 Tn, the circadian rhythm gene Per1 suppresses the expression of Th1 cytokines by inhibiting Akt/mTORC1 signal transduction [99], shifting the balance of helper T cell subsets towards Th2 and increasing the level of secreted antibodies during stress response [98].

Among T cells, Th17 is influenced by circadian rhythms. Th17 is a group of CD4^+^ helper T cells that play a vital role in immune response and are related to inflammatory reactions and autoimmune diseases. It secretes cytokines such as IL-17 and IL-22 and its differentiation and function depend on the transcription factor RORγt [100]. Genes *Nfil3* and *Rorc* play a key role in Th17 differentiation [101]. NFIL3 expression is regulated by REV-ERBα and REV-ERBβ [102]. Expression levels of NFIL3 and RORγt were measured in a study, showing opposite patterns during day and night [101]. The study also found increased Th17 cells in the intestine and spleen of NFIL3 knockout mice (Nfil3^−/−^) and inhibition of Th17 differentiation with NFIL3 overexpression [101].

Circadian rhythms have a significant impact on Th17 cells beyond CD4 Tn cells. A study found that circadian rhythm disorder (CRD) contributes to the development of allergic rhinitis (AR) by affecting Th2 and Th17 immune responses [103]. Levels of IL-4, IL-6, IL-13, and IL-17A significantly increased in CRD^+^AR patients, while IFN-γ levels decreased [103]. In addition, levels of Th2 and Th17 cells were upregulated, while Th1 and Treg cells were downregulated [103]. These findings suggest that CRD enhances the immune response mediated by Th2 and Th17 in AR by regulating Th1/Th2 and Th17/Treg immune balance [103]. Therefore, changes in clock activity can increase susceptibility to allergic diseases.

In CD4 T cells, the circadian rhythm gene Per1 inhibits Akt/mTORC1 signaling, thereby suppressing the expression of Th1 cell cytokines and promoting the differentiation of CD4 T cells towards Th2 cells. This results in a tendency for CD4 T cells to produce more Th2 cells at specific time points and an increase in antibody levels under stress stimuli. In addition, the circadian rhythm also plays a significant role in Th17 cells in helper T cells. The circadian rhythm genes Nfil3 and Rorc play crucial roles in the differentiation of Th17 cells, and the quantity and functionality of Th17 cells may vary at different time points. Circadian rhythm disruption (CRD) can also affect the immune response of Th2 and Th17 cells, leading to excessive activation of these cells and increased susceptibility to allergic diseases. In summary, the circadian rhythm regulates the differentiation, function, and immune response of T cells by modulating specific circadian rhythm genes and signaling pathways, thus influencing the balance and function of the immune system.

### NK cells

Natural killer (NK) cells are innate immune cells that directly eliminate tumor cells without activation. They release granzyme B, perforin, and pro-inflammatory cytokines, such as TNF-α [104, 105]. NK cells also regulate other white blood cells, including macrophages, T cells, and dendritic cells (DCs), through cytokine release [106]. Circadian rhythms affect NK cell function, including cytokine release and cytolytic factors [107], but their impact on NK cell development and function remains unclear.

A recent study investigated the impairment of NK cell function and potential mechanisms due to circadian rhythm disorders [108]. Using a chronic shift-lag mouse model, researchers observed decreased NK cell numbers in the spleen and lungs. They also found an increase in aging NK cells (CD27^−^CD11b^+^) and a decrease in functional NK cells (CD27^+^CD11b^+^) in the bone marrow and spleen. These findings suggest that circadian rhythm disorders promote NK cell apoptosis and aging, affecting their quantity and function [108]. In addition, disrupted circadian rhythms led to changes in NK cell receptor expression, with increased immature receptors (CD117 and CD127) and decreased functional receptors (Ly49D, Ly49G2, and Ly49H) [108].

**Fig. 2:**
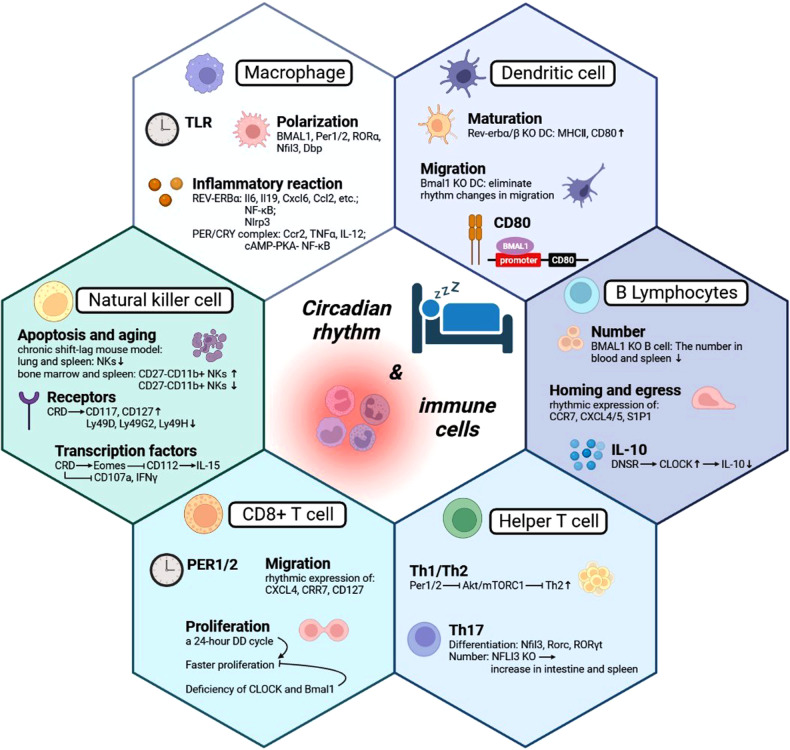
The effect of circadian rhythm on immune cells. Circadian rhythm can influence immune cell function and response by modulating specific genes and signaling pathways, thereby affecting the balance and functionality of the entire immune system. In macrophages, circadian rhythm can affect Toll-like receptor activation and downstream signaling pathways, regulating immune activity and inflammatory response. Additionally, circadian rhythm can impact macrophage polarization, metabolic pathway selection, and other factors, thus affecting their ability to participate in inflammation and immune function. For dendritic cells, circadian rhythm can affect their maturation, antigen presentation, and migration functions. As for B cells, their development and migration can be influenced by circadian rhythm, but the underlying mechanisms require further research. In T cells, circadian rhythm can affect their migration, proliferation, and differentiation, thereby impacting their immune function and anti-tumor response. In Th17 cells, circadian rhythm can influence the expression of key genes such as NFIL3 and RORγt, affecting Th17 differentiation and function. Finally, circadian rhythm can also affect the number and function of NK cells, thus influencing their ability to clear tumor cells.

Circadian rhythm disorder significantly reduces the secretion of CD107a and IFN-γ by NK cells, impairing their ability to clear MHC-I deficient tumor cells and B16 melanoma cells [108]. IL-15 signal transduction is crucial for NK cell survival and homeostasis in the peripheral immune environment [109]. CD122 plays a key role in maintaining IL-15 stimulation [110]. In NK cells with circadian rhythm disorders, the expression of Eomes and T-bet, important transcription factors that regulate NK cell development and function, is altered [108]. Eomes affects NK cell development and function by transcriptionally regulating CD122, and its expression is significantly reduced in NK cells from mice with chronic shift-lag. Blocking CD122 abolishes the inhibitory effect of chronic shift-lag on CD107a and IFN-γ secretion by NK cells [108], indicating that circadian rhythms indirectly affect IL-15 levels through the regulation of Eomes and CD122 expression, thereby impacting NK cell immune monitoring ability (Fig. 2).

In general, disruption of the circadian rhythm has a negative impact on NK cell function. Research has found that circadian disruption leads to a decrease in the number of NK cells, an increase in aging NK cells, and affects the expression of NK cell receptors. In addition, circadian disruption reduces the immune surveillance ability of NK cells and their ability to clear tumor cells. These effects may be achieved through the regulation of key transcription factors and signaling pathways. Further research is helpful in gaining a deeper understanding of the relationship between the circadian rhythm and the development and function of NK cells.

## The sleep-wake cycle is involved in influencing immune system function and DNA damage repair, and is regulated by inflammation and cytokine feedback

As one of the three major cycles that affect mammalian physiological activity and function, the sleep-wake cycle plays an important role in maintaining normal physiological functions and ensuring normal gene expression. Especially for the immune system, sleep, as a pathway for specifically regulating the endocrine and autonomic nervous systems, should systematically control immune functions [111, 112]. Human studies have confirmed that regular sleep has a promoting effect on the primary immune response [113, 114], so maintaining a regular sleep-wake cycle can promote the formation of a good immune system in the body.

### Sleep affects the production of cytokines and participates in the regulation of inflammation

#### Sleep affects immune status by regulating the secretion of cytokines by T cells

The cytokine balance between helper T cell 1 (Th1) and helper T cell 2 (Th2) is crucial for immune response. Th1 cells release interferon-γ (IFN-γ), interleukin-2 (IL-2), and tumor necrosis factor-α (TNF-α), promoting cellular (type 1) reactions against intracellular pathogens. Th2 immunity (IL-4, IL-5, IL-10, and IL-13) stimulates humoral (type 2) defense against extracellular pathogens [115]. Nocturnal sleep has been reported to benefit the transition to Th1-mediated immune defense. IFN-γ and IL-10 production in vitro exhibit a circadian rhythm, with a peak at around 03:00 during nighttime sleep [116, 117]. Sleep, particularly in the early stages dominated by slow-wave sleep (SWS), inhibits glucocorticoid release and promotes growth hormone (GH) and prolactin release [118–120]. These factors shift the Th1/Th2 balance towards Th1 advantage, promoting cell-mediated immune responses against intracellular pathogens [121, 122]. Another study confirmed that nocturnal sleep inhibits Th2 cell production compared to wakefulness, while the effect on Th1 cells remains unclear. Thus, sleep induces a shift towards Th1 dominance [115]. Inhibition of cortisol release and increased melatonin levels at night may contribute to Th1 dominance [116, 123]. However, this effect is limited to early sleep, mainly SWS, and reversed during late sleep dominated by rapid eye movement (REM) sleep, with lower CD4^+^ cell ratio of IFN-γ/IL-4 during sleep compared to wakefulness [115]. Elderly individuals with sleep and SWS disorders show reduced GH and prolactin release, leading to Th2 dominance [124, 125].

Besides helper T cells, sleep also decreases TNF-α production by T suppressor/cytotoxic (CD8^+^) cells [115]. This decrease may be related to effector CD8^+^ cells and memory T cells rather than initial CD8^+^ cells, reflecting changes in migration to extravascular tissues [126, 127]. However, the role of cell recycling in sleep-mediated immune function remains unclear.

The impact of sleep on the immune system is mainly reflected in cytokine balance and the number of immune cells. Sleep can promote the activation of Th1 cells, increase the production of IFN-γ and other related cytokines, thereby enhancing the ability to combat intracellular viruses and bacteria. At the same time, sleep can inhibit the release of cortisol, promote the release of growth hormone and prolactin. These factors work together to tilt the Th1/Th2 cytokine balance towards Th1 dominance, thereby enhancing cell-mediated immune responses. In addition, sleep can also suppress the production of Th2 cells, reduce the production of IL-4 and other related cytokines by CD4^+^ cells, further strengthening the Th1 dominance. Furthermore, sleep can also reduce the production of TNF-α by CD8^+^ cells. Although the specific mechanisms are not fully understood, the effects of sleep on the immune system primarily occur during the early stages of sleep (dominated by slow-wave sleep), while rapid eye movement (REM) sleep may have opposite effects. In elderly individuals, sleep and slow-wave sleep disorders lead to a reduction in the release of GH and prolactin, and the cytokine balance tilts towards Th2 dominance. In conclusion, sufficient sleep helps maintain the normal function of the immune system, especially enhancing cell-mediated immune responses. However, further research is still needed to understand the impact of sleep on immune function.

#### Peripheral blood and intestinal inflammation are regulated by clock genes

Inflammation is linked to sleep, with various inflammatory cytokines involved in the development of cardiovascular diseases, diabetes, and other conditions. Sleep has an impact on the activities of these cytokines [128]. Epidemiological data suggest that insufficient sleep can increase the risk and mortality of chronic diseases due to its influence on the inflammatory mechanism [129]. Experimental sleep deprivation studies have reported alterations in immune response, leading to elevated levels of inflammatory markers such as IL-6, TNF-α, and C-reactive protein [130–132]. The cellular origin of pro-inflammatory cytokine activity remains unclear, although monocytes are considered the main contributors in peripheral blood [128]. TLR4-mediated lipopolysaccharide ligation has been shown to significantly increase IL-6 and TNF-α production in peripheral blood monocytes following sleep deprivation [128]. Moreover, insufficient sleep can induce changes in the expression of pro-inflammatory cytokines, the circulating clock gene PER1, immediate early response genes, as well as signal transduction mediators and growth factor-related genes. Structural functional bioinformatics analysis has revealed the involvement of multiple signaling pathways, including the nuclear factor κB inflammatory signaling system and classic hormone and growth factor response pathways, in the transcriptional response of white blood cells to sleep deprivation [128].

The biological clock plays a crucial role in regulating intestinal immunity and maintaining intestinal barrier function. For instance, knockout of BMAL1 disrupts the homeostasis of gut group 3 innate lymphoid cells (ILC3), leading to impaired intestinal epithelial reactivity, microbiome imbalance, increased susceptibility to intestinal infection, and lipid metabolism disorders [133]. BMAL1 knockout mice also exhibit reduced small intestinal crypts and impaired intestinal regeneration after radiation-induced gastrointestinal syndrome [134], highlighting the importance of BMAL1 in intestinal cell proliferation. Overexpression of CRY1 inhibits vascular inflammation induced by sleep deprivation (SD), possibly through the NF-κB and cyclic adenosine monophosphate/protein kinase A (cAMP/PKA) pathways in vivo [135]. Acute sleep deprivation (ASD) commonly affects shift workers, causing lethargy and persistent fatigue. ASD contributes to inflammation by influencing the intestinal microbiota. In elderly individuals with insomnia, the impact of physical activity on sleep disorders can be regulated by the gut microbiota’s primary metabolites. The genus Monoglobus shows a negative correlation with physical activity and sleep latency, while exhibiting a positive correlation with sleep efficiency [136]. Disruption of circadian rhythm leads to decreased abundance of specific bacteria (e.g., Lactobacillus and Ruminococcus) along with downregulation of BMAL1 [137]. Increased Candidatus Arthromitus in the SD group may contribute to colitis, intestinal barrier dysfunction, and systemic inflammation [138]. Thus, circadian rhythm and microbiota composition play crucial roles in maintaining intestinal barrier function. Adequate sleep and self-regulation of the microbiota might have protective effects against disease and help restore the intestinal epithelial barrier, thereby improving inflammation [138].

In summary, it can be found that sleep regulates cytokine secretion and intestinal microflora through various pathways, affecting the inflammatory state of the body, thereby participating in the development of various inflammatory diseases. Breaking the regular sleep-wake cycle may increase the secretion of pro-inflammatory cytokines in the peripheral blood, thus increasing the risk of cardiovascular disease, diabetes, etc. In addition, the downregulation of specific flora abundance and clock gene expression caused by acute sleep deprivation may lead to colitis, intestinal barrier dysfunction, and other diseases.

Sleep has a significant impact on the immune system and inflammatory state. Lack of sleep can lead to increased inflammatory response, increased risk of chronic diseases, and mortality rates. Experimental sleep deprivation can alter immune responses and increase circulating levels of inflammatory markers. Sleep deprivation also affects changes in the expression of pro-inflammatory cytokines and signaling pathways. The biological clock plays a crucial role in intestinal immunity and barrier function. Knocking out the BMAL1 gene disrupts the homeostasis of intestinal group 3 innate lymphoid cells, leading to impaired intestinal responsiveness and dysbiosis. Adequate sleep and self-regulation of the gut microbiota can protect the immune system, improve inflammatory status, and restore intestinal barrier function. Therefore, sleep has a significant impact on the body’s inflammatory state and the development of inflammatory diseases, and disrupting the circadian rhythm may increase the risk of various diseases.

### Sleep deprivation mediates the occurrence of cancer via affecting DNA damage repair and oxidative stress

Circadian rhythm disorders caused by chronic imbalances between behavioral rhythms and external light/dark cycles are common [139] and have been proven to be causes of various cancers [140] and chronic health problems [141]. Increasing evidence suggests that circadian rhythms affect multiple pathways regulating cancer outcomes, including cell cycle checkpoints, apoptosis, cell proliferation, DNA repair, and inflammation [142–150].

Circadian rhythm disorders are prevalent among night shift workers [151], leading to severe disruption of human biomolecular pathways during and after night shifts [152–155], thus increasing the cancer risk [156]. Rodent studies confirm this view, showing that changes in circadian rhythms can disrupt cell cycles, alter metabolism, promote tumor development, induce tumor cell growth, and trigger tumor-related immune cell remodeling under simulated shift work and chronic jet lag conditions [157–160].

A study analyzed volunteers subjected to simulated day or night shift schedules, which resulted in circadian rhythm disorders, and collected blood samples to study their impact on endogenous biological processes [139]. The findings revealed impaired effectiveness of repairing DNA damage caused by endogenous sources and exogenous irradiation in white blood cells obtained under night shift conditions. Moreover, compared to the simulated night shift group, night infrared (IR) exposure led to more DNA damage in the white blood cells of the simulated night shift group [139]. In addition, it reports for the first time the circadian rhythm imbalance of cell cycle and DNA repair regulatory factors in human leukocytes after night shifts. Key genes involved in cell cycle checkpoint activation and DNA repair were identified, including ATR, CDK4, CDKN1C, TP53, WEE1, ERCC6, TP53, RPA3, XPA, H2AFX, PARP1, and RAD50. Disruption of the circadian rhythm of these genes’ expression suggests a direct link between cellular circadian rhythm mechanisms and the carcinogenic risk of night shift work [139].

**Fig. 3:**
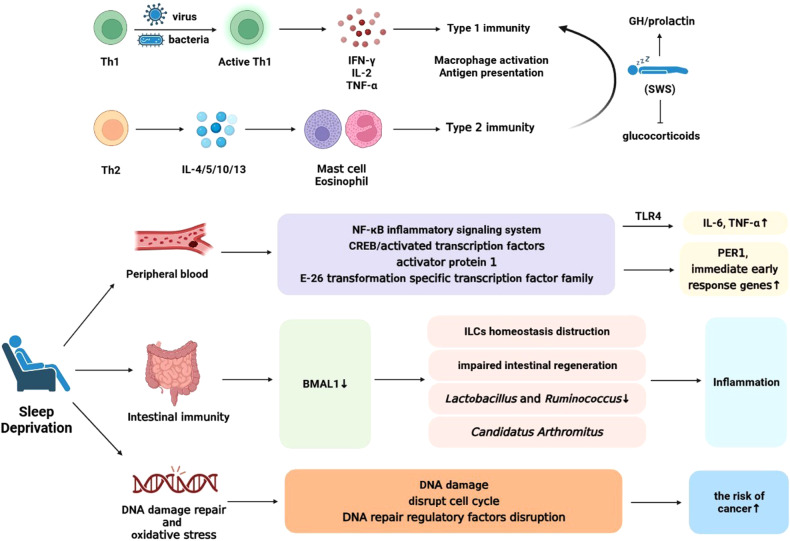
The sleep-wake cycle is involved in affecting immune system function and DNA damage repair. Nighttime sleep, particularly in the early stages characterized by slow-wave sleep (SWS), promotes a shift towards Th1-mediated immune defense by inhibiting glucocorticoid release and promoting the release of growth hormone (GH) and prolactin. Sleep also inhibits Th2 cell production, further reinforcing Th1 dominance. Circadian rhythms regulate the secretion of cytokines and gut microbiota, significantly impacting the body’s inflammatory state. Sleep deprivation leads to abnormal cytokine secretion, altering the inflammatory state. Experimental sleep deprivation studies have shown increased immune response and elevated levels of inflammatory markers such as IL-6, TNF-α, and C-reactive protein in circulation. Monocytes are considered a primary factor in peripheral blood inflammation. Additionally, sleep deprivation affects the expression of inflammatory cytokines, circadian clock gene PER1, signaling molecules, and growth factor-related genes. Sleep is crucial for maintaining normal immune function, particularly enhancing cell-mediated immune responses. Furthermore, sleep deprivation disrupts circadian rhythms, impairs DNA damage repair, and increases the risk of cancer occurrence. Night shift workers often experience disrupted circadian rhythms, which interfere with cellular processes involved in DNA repair, cell cycle regulation, and inflammation, thereby increasing cancer risk. Studies have shown that sleep deprivation impairs the effective repair of DNA damage from both endogenous and exogenous sources. Moreover, disruptions in the expression of key genes involved in cell cycle checkpoints and DNA repair further indicate a direct link between circadian rhythm disturbances and the carcinogenic risk associated with night shift work. Severe sleep deprivation may exacerbate these effects, potentially leading to premature death in model organisms. Insufficient sleep not only affects brain function but also disrupts the body’s antioxidant response, leading to increased oxidative stress and DNA damage.

Severe sleep deprivation can have even more serious consequences, leading to premature death in model organisms such as dogs, rats, cockroaches, and flies [161–164]. Insufficient sleep impairs brain function [165–167], and some propose that sleep’s function is to prevent oxidative stress in the brain [168]. Studies have reported changes in antioxidant response during sleep deprivation [169–173]. Insufficient sleep can result in ROS accumulation due to increased production, decreased elimination, or both [174]. While most ROS is generated during mitochondrial oxygen-dependent ATP synthesis [175], it is also produced as a by-product of oxidative protein folding [176]. Lack of sleep can induce endoplasmic reticulum stress, leading to oxidative stress and increased demand for protein folding, thereby increasing ROS production [177–179]. However, many studies have not detected significant oxidative damage in the brain during sleep deprivation [180–183], potentially due to effective activation of antioxidants and cellular protective mechanisms [170, 184–188]. Notably, gastrointestinal diseases are often associated with sleep restriction and abnormal ROS levels, which may contribute to tumor development through mechanisms like DNA damage and inflammation [189–196] (Fig. 3).

Factors that primarily contribute to the disruption of circadian rhythm include insufficient sleep, night shift work, and sleep deprivation. These factors can lead to disturbances in DNA damage repair and oxidative stress, increasing the risk of cancer. Sleep deficiency can interfere with the circadian rhythm, affecting multiple pathways such as cell cycle, apoptosis, cell proliferation, DNA repair, and inflammation, thereby influencing cancer outcomes. Night shift workers commonly experience circadian rhythm disruption, which increases their susceptibility to cancer. Animal studies have also shown that alterations in circadian rhythm can disrupt cell cycle, alter metabolism, promote tumor development, and induce tumor cell growth and immune cell reshaping under simulated night shift and long-term jet lag conditions. Research has also found circadian rhythm disruption in genes involved in DNA repair pathways among volunteers following a simulated night shift schedule. The effectiveness of the DNA damage repair process is compromised in white blood cells obtained during night shift work. The circadian rhythm of cell cycle and DNA repair regulatory factors in human leukocytes is also imbalanced after night shifts, and these genes are crucial for identifying and repairing DNA damage. Severe sleep deprivation may also lead to premature death in model organisms, possibly due to impaired brain function. During sleep deprivation, changes occur in the brain’s antioxidant response, and insufficient sleep can result in the accumulation of reactive oxygen species (ROS), leading to oxidative stress. Considering the pathological implications associated with sleep restriction, such as gastrointestinal disorders, abnormal levels of ROS may contribute to tumorigenesis through various mechanisms.

### Inflammation, cytokines, and immune cells can provide feedback to regulate sleep

Sleep and immunity have a bidirectional relationship. Sleep affects the immune system, and changes in the immune system can also affect sleep. Diseases characterized by low-level inflammation have been identified, including degenerative diseases, cardiovascular diseases, metabolic disorders, and chronic pain [197–199]. Inflammatory disorders are considered a potential mechanism linking sleep time and mortality [200, 201].

In a 7-year study of over 2500 elderly men, short sleep time (5 h) was associated with an increased risk of all-cause mortality due to increased inflammatory burden (i.e., composite measurement of CRP, IL-6, TNF-α, sTNF-RII, IFN-γ levels); adjusting for the inflammatory burden significantly weakens this relationship, indicating that the inflammatory mechanism disrupts the relationship between sleep time and mortality [202]. Elevated CRP has been recognized as a marker for predicting cardiovascular risk [203], suggesting that inflammation may be the basis for the association between sleep deprivation and mortality, as well as the association between sleep deprivation and increased cardiovascular disease risk [204].

In most studies, inflammatory status is measured by levels of CRP or IL-6, and higher levels of CRP and IL-6 are associated with reduced sleep time [205]. The mediating effect of inflammation seems to be unique to short sleep duration, as other sleep characteristics such as sleep latency or nocturnal wakefulness are not associated with the burden of inflammation. Elevated inflammatory markers have also been reported in prolonged sleep (8 or 9 h) [206–210], although uncontrolled disease processes may mediate this relationship. However, some studies have not found an association between inflammatory markers and sleep time [211–215], increasing the heterogeneity of research results.

Regarding inflammatory cytokines, poor subjective sleep quality has been associated with higher production of stimulating IL-1, TNF-α, and IL-6 in a cross-sectional study [213]. Recently, poor sleep quality in postmenopausal women has been associated with increased IL-6 responsiveness after psychological and social stress [216]. In terms of immune cells, an increase in white blood cell count was associated with lower sleep efficiency and higher nocturnal awakening time in women [217–220].

In summary, the bidirectional relationship between sleep and immunity is supported by research results. Inflammatory markers, cytokines, and immune cells are connected to sleep, and their dysregulation may be a potential mechanism for sleep disorders affecting disease risk [221]. Changes in sleep during illness, such as infection, may have implications for recovery [222].

## Effect of circadian rhythm on tumors and their microenvironment

A regulatory mechanism exists between circadian rhythms and cancer genes, which is relevant to tumor incidence and malignancy. Gene rhythms associated with metabolism and endocrine function significantly contribute to tumor development. Abnormal biological rhythm disrupts the body’s innate and acquired immunity, promoting malignant tumor progression. Studies have shown that in the malignant thyroid nodule group, the expression level of CLOCK and BMAL1 was significantly higher, while the expression level of CRY2 was lower [223]. The infiltration of immune cells such as macrophages and neutrophils in renal clear cell carcinoma correlates with rhythmic changes in the expression of CLOCK-related components (BMAL1, PER, etc.) [224, 225]. CLOCK also participates in regulating the microglial content of glioblastoma stem cells (GSC) in glioblastoma (GBM) through transcriptional regulation of chemokines [226]. Single-cell transcriptomic analysis has revealed an association between circadian rhythm disturbance (CRD) and poor prognosis as well as drug resistance in lung adenocarcinoma. CRD score was significantly associated with poor prognosis and resistance to anti-cancer treatments, including chemotherapy and tyrosine kinase inhibitor (TKI) therapy, in LUAD patients [227]. Disruption of clock-related genes promotes colorectal cancer in a model of gut organoids, and loss of circadian rhythm was observed in patient-derived organoids [228].

The cell cycle and biological clock function as biological oscillators, and there is increasing evidence of molecular connections between these two oscillators using various coupling mechanisms, which are often misregulated in cancer cells [146]. The cell cycle consists of four stages: G1, S, G2, and M phases, with the G0 phase representing a resting state when cells temporarily stop dividing. Key cell cycle molecules (cyclins) regulate cell cycle progression in a sequential and unidirectional manner [229], and the kinase activity of cyclin-CDK complexes triggers specific events at defined times in the cell cycle [230]. The biological clock acts as a biological oscillator with conceptual and mechanical similarities to the mechanisms controlling cell division. Both the cell cycle and biological clock exhibit sequential stages of transcription/translation, protein modification, and degradation [146]. Their interaction jointly regulates tumor cell activity. A deeper understanding of the molecular links between the cell cycle and circadian clock may serve as the foundation for designing innovative therapeutic strategies.

The tumor microenvironment (TME) significantly influences tumor development. TME usually consists of the extracellular matrix (ECM) and various cells including congenital myeloid cells [such as tumor-associated macrophages (TAM), dendritic cells, and myeloid suppressor cells (MDSC)], lymphocytes [such as T cells and natural killer (NK) cells], and endothelial cells [231]. Understanding the molecular aspects of the circadian clock in tumorigenesis and the symbiotic interaction between cancer cells and the TME is currently a major focus of cancer research [232]. Recent evidence indicates that circadian rhythm disturbances are associated with increased cancer risk and promote metastasis in multiple tumor types. The biological clock also plays a regulatory role in tumor growth and several cancer markers. Furthermore, studies have confirmed that the circadian rhythm regulates immune function, which is of great significance in cancer treatment since the immune system plays a crucial role in eliminating and suppressing malignant tumors.

### Circadian rhythm participates in the development of tumors by affecting various kinds of cells

The concentration of immune-mediated hormones and cytokines fluctuates rhythmically throughout the day [148]. Any disturbance in the balance between anti-tumor immune cells and pro-tumor immunosuppressive cells may enable cancer cells to escape immune control, resulting in disease occurrence and development [233]. T cells and myeloid-derived suppressor cells (MDSCs) play a major role. CD8^+^ T cells are responsible for selectively killing tumor cells [234], whereas MDSCs inhibit immune response and limit inflammation, reducing the ability of T cells to kill cancer cells [235]. External disturbance of circadian rhythm in mice tumors leads to faster tumor growth due to the interruption of internal circadian rhythm of cancer cells, amplifying their proliferation [236]. Interruption of central circadian rhythm promotes tumor growth by affecting non-cancer cell signaling, while MDSC accumulation inhibits tumor-specific immune response [236]. Adrenergic signaling pathway disruption increases MDSC accumulation, regulating hematopoietic stem cells and CXCL12 expression periodic fluctuation [237], and impacting cytokine expression and cytolytic function of NK cells [238].

TAM is the most significant subgroup in TME, influencing tumor growth and metastasis [239]. BMAL1 depletion in macrophages regulates pro-inflammatory cytokine production through HIF1α upregulation, ROS accumulation, and NRF2 downregulation [240, 241]. The balance between BMAL1 and HIF1α affects macrophage metabolism and anti-tumor immunity, and BMAL1 knockout increases septic mice mortality [242, 243]. Co-injection of cancer cells and Bmal1 KO macrophages promotes tumor growth with reduced CD8^+^ T cell infiltration [240]. Activation of REV-ERBα inhibits CCL2 and its downstream signals, suppressing macrophage adhesion and migration [244]. GAMs divide into M1 and M2 subtypes with opposite functions in glioma progression [245–247]. M2 subtype promotes tumor proliferation and metastasis by reducing BMAL1 expression in TAM and regulating tumor-related protein expression and cell apoptosis [248]. Leukocyte migration, crucial in tumor development, is regulated by the biological clock [249]. Bmal1 deletion in endothelial cells eliminates time difference in migration-promoting molecule expression [250], while CLOCK overexpression increases leukocyte adhesion to endothelium via upregulating ICAM-1, VCAM-1, and other molecules [251]. CRY1 overexpression in endothelial cells inhibits inflammatory cytokines (IL-1β, IL-6, TNF-α), adhesion molecules (VCAM-1, ICAM-1, e-selectin), and NF-κB pathway activation, impairing monocyte adhesion [135].

The circadian clock regulates cancer cell products, TME, and their symbiotic interactions. Breast cancer and melanoma mouse models show that circadian rhythm disorder enhances cancer cell proliferation, spread, stemness, and metastasis, induces immunosuppressive TME by increasing TAM and regulatory T cell (Treg) proportion, and promotes differentiation of macrophages into an anti-inflammatory phenotype resulting in reduced infiltration of active CD8^+^ T cells [160, 252]. RORγ activation inhibits tumor growth, prolongs survival by enhancing Th17 cell differentiation and function, and reducing Treg levels [253]. RORα activation maintains cholesterol metabolism balance in CD8^+^ T cells, enhancing their activity through weakening the NF-κB pathway [254].

### There is molecular coupling between cell cycle and biological clock, and they are jointly involved in the occurrence and development of cancer

DNA replication and cell division require a large number of bioenergy components [255]. Their synthesis is regulated by a clock-dependent mechanism, indicating that the demand for cell cycle biosynthesis is controlled by the circadian rhythm of the metabolic cycle and environmental supply [256, 257]. Mice carrying core clock gene-specific ablation have shown the importance of functional clocks for the normal mitotic rhythm and progression of progenitor cells and/or stem cells in various cell types [258–262]. The circadian clock also plays a role in coordinating the regeneration process after inducing damage to the skin [263] and intestinal epithelium [261].

The circadian clock is not a mandatory component of the cell cycle but contributes to the timing of cellular events and adaptive mechanisms needed for optimal tissue function [146]. There is a significant relationship between the circadian clock and the cell cycle, with evidence of circadian rhythm changes in cyclin abundance and clock gene expression at specific cell cycle stages [264, 265]. The circadian clock controls the expression of CDK and cyclins such as CDK2, CDK4, and cyclin D1, as well as cell cycle inhibitors including Wee1, p21, p27, and p57 [266]. CLOCK-BMAL1 regulates the circadian expression of the G2/M inhibitor WEE1 [267], while the expression of p21 is regulated by antagonistic effects of ROR and REV-ERB proteins [268]. NONO, in association with PER protein, regulates the circadian rhythm regulation of p16 expression [263]. P53 directly binds to the Per2 promoter and inhibits CLOCK-BMAL1-mediated transcription [269].

Cell cycle checkpoints detect replication stress or DNA damage and delay cell cycle progression to repair or eliminate damaged cells. Circadian clock proteins interact with checkpoint reactive protein complexes to mediate DNA damage responses [146]. This coupling may protect cells from DNA damage caused by ultraviolet radiation by limiting replication during dark periods and optimizing repair mechanisms during the day [259, 270]. DNA damage can also regulate circadian rhythms and affect cell cycle progression [271].

Cell cycle disruption is a key event in carcinogenesis, and there is a correlation between cancer incidence rate and circadian rhythm disorder [272]. Both cell cycle genes and biological clock genes are incorrectly regulated in cancer cells [266]. Exploring the interaction between circadian rhythm and the cell cycle in cancer is of great significance. However, the causal relationship between sleep circadian rhythm disorders and cancer emergence and progression, the effectiveness of controlling cancer with the circadian clock, and the impact of cancer-related therapies on the biological clock remain unresolved [146].

### Metabolism and multiple factors in tumor microenvironment are regulated by biological rhythm

Apart from that, tumor-induced signals such as granulocyte-macrophage colony-stimulating factor (GM-CSF), granulocyte-CSF (G-CSF), macrophage-CSF (M-CSF), stem cell factor (SCF) and vascular endothelial growth factor (VEGF), along with inflammatory signals including IFN-γ, IL-1β, IL-6, and TNF-α, are rhythmically expressed and released by specific cells [236]. Central circadian rhythm changes can increase the production of these signal factors in tumor cells. Knockout of clock proteins Cry1 and Cry2 leads to a constitutive increase in pro-inflammatory cytokines via NF-κB and PKA signals, indicating direct regulation of cytokine expression by circadian genes [75]. Deletion of myeloid-specific circadian rhythm gene disrupts chemokine expression rhythm and Ly6Chi monocyte tissue recruitment [242]. Adhesion molecules and chemokines in endothelial cells oscillate in a circadian rhythm, affecting leukocyte adhesion and exosmosis [273].

Clock components regulate the expression of angiogenesis factors in cancer cells, such as HIF1α, ARNT, and VEGF, promoting tumor growth and metastasis [56, 274–276]. CLOCK knockout decreases angiogenesis factor expression in human colorectal cancer (CRC) cells, while CLOCK overexpression increases it [274]. Xenotransplantation tumors show fluctuating VEGF levels controlled by PER2 and CRY1 transcription in a circadian rhythm [277]. Clock genes not only regulate the circadian rhythm and transcription factor activity of cancer cells but also affect macrophage, neutrophil, dendritic cell, and lymphocyte infiltration [224, 225]. Clock gene mutations in cancer patients lead to genomic instability, T cell depletion, upregulation of immunosuppressive molecules, and immune escape [97, 278].

In a study on breast cancer, chronic jet lag (JL) was shown to impact its development [252]. JL mice had higher blood lipid levels and increased leptin and insulin resistance, which are related to weight gain, obesity, and type 2 diabetes [252, 279, 280]. Expression of Per2 and Cry2 clock genes decreased in primary tumors of JL mice, with significant changes in genes involved in light transduction in the tumors and mononuclear myeloma cells [252]. CRD-related enhancement of tumor genesis and metastasis involved the CXCL5-CXCR2 axis. CRD increased Cxcl5 expression, leading to increased infiltration of CXCR2^+^ myeloid cells (such as MDSCs) [252]. This promoted the accumulation of MDSCs, TAM, and TAN, creating an immunosuppressive microenvironment [281]. These cells directly inhibit T-cell response and suppress CD8 T-cell infiltration, impairing anti-tumor activity [281–284]. Inhibition of CXCR2 significantly reduced lung metastasis and bone diffusion in JL mice [252]. The CXCL12-CXCR4 axis also plays a crucial role in JL mouse cancer cell metastasis and diffusion [252]. Its control of circadian rhythm and its involvement in immunosuppression and breast cancer metastasis have been extensively described [237, 250, 285–288] (Table 1).

**Table 1:** Clock genes participate in the development of tumors.

Clock gene	Cell	Effects	Ref.
BMAL1↓	Macrophage	↑ HIF1α, ROS; ↓ NRF2→ ↑ pro-inflammatory cytokines→ ↑ the mortality of septic mice	[71, 72]
	GAM (M2 subtype)	↑ tumor-related proteins; ↓ cell apoptosis→ ↑ tumor proliferation and metastasis	[81]
	Endothelial cell	Time difference in the expression of migration-promoting molecules was eliminated	[83]
CLOCK↑	Endothelial cell	↑ ICAM-1, VCAM-1→ ↑ the adhesion of leukocytes to endothelium	[84]
	CRC cells	↑ angiogenesis factors	[91]
CRY↓	Endothelial cell	↑ inflammatory cytokines, adhesion molecules, NF-κB pathway→ ↑ monocyte adhesion	[85]
	Tumor cell	↑ NF-κB, PKA→ ↑ pro-inflammatory cytokines	[32]
RORα↓	CD8^+^ T cell	↑ NF-κB pathway → ↓ CD8^+^ T cell activity	[89]
RORγ↓	Th17 cell, Treg	↓ differentiation and function of Th17 cells; ↑ the level of Tregs → ↑ tumor growth	[88]
REV-ERBα↑	Macrophage	↓ CCL2 → ↓ the adhesion and migration	[75]

## Summary

Circadian rhythm is a widespread physiological phenomenon in nature. It has been increasingly recognized for its impact on the body’s physiology and pathology. Maintaining circadian rhythm is crucial for normal cellular function and metabolic pathways [1]. Disruption of circadian rhythms, such as through shift work, can alter clock gene expression and increase disease risk [63].

In pathological conditions like cancer and allergic diseases, abnormal clock gene expression affects the phenotype and function of immune cells, including macrophages [63], dendritic cells [81, 289], and lymphocytes [1, 90, 98, 102, 103]. It also influences cytokine and chemokine levels, contributing to the development of various diseases.

Circadian rhythm controls Toll-like receptor expression, polarization, and cytokine secretion in macrophages through BMAL1, REV-ERBα, and PER/CRY complex [63]. Disruption of Bmal1 causes migration defects in dendritic cells and affects CD80 expression timing, impacting CD8^+^ T cell proliferation [81, 289]. BMAL1 also regulates chemokine receptor secretion in B cells, while CLOCK negatively correlates with IL-10 levels in B10 cells, influencing CD4^+^ T cell and Tr1 cell production [1, 90]. Circadian oscillation of proliferation and migration has been observed in CD8^+^ T cells, indicating their endogenous circadian rhythm [91]. Furthermore, Th17-related genes Nfil3 and Rorc play a role in disease regulation through immune response mediation [102].

The sleep-wake cycle significantly influences mammalian physiological activities and functions. It affects cytokines, inflammation, DNA damage repair, and oxidative stress [115]. Sleep can promote the transition to Th1-mediated immune defense and inhibit cortisol release, leading to increased melatonin levels [115]. Lack of sleep alters pro-inflammatory cytokine expression and disrupts signal transduction pathways [128]. Clock genes also regulate intestinal inflammation by affecting the abundance of specific gut bacteria through BMAL1 downregulation [138]. Shift work-induced sleep-wake cycle disorders impact circadian rhythms in white blood cell cycle and DNA repair pathway-related genes [139]. This disruption induces endoplasmic reticulum stress, oxidative stress, and increased ROS production, potentially causing premature biological death [174]. Inflammation, cytokines, and immune cells reciprocally regulate sleep. Inflammatory mechanisms disrupt the relationship between sleep duration and mortality, potentially contributing to increased cardiovascular disease risk [221].

Circadian rhythm is associated with cancer incidence and malignancy. Rhythmic genes influence the expression of cancer genes, metabolism, and endocrine functions, contributing to the development of glioblastoma, breast cancer, and other tumors [243]. Clock gene mutations affect various cells in the tumor microenvironment, promoting immune escape, tumor growth, and altering the balance between anti-tumor immune cells and pro-tumor immunosuppressive cells. They also regulate TAM cell subsets’ proliferation, migration, infiltration ability, Th17 and Treg levels, and CD8^+^ T cell numbers [243]. Molecular coupling exists between the cell cycle and the biological clock. Clock genes are expressed at specific stages of the cell cycle, resulting in rhythmic changes in cyclin abundance. Proteins encoded by rhythmic genes, such as BMAL1, CLOCK, PER, regulate cell cycle-related genes like Wee1, p21, p16, and p53. Circadian clock proteins directly interact with checkpoint reactive protein complexes, mediate DNA damage responses, and impact cancer development [146]. In addition, clock genes regulate cytokines, chemokines, and migration-promoting molecules produced by tumors, playing a role in immunosuppression and cancer metastasis [243]. Targeting clock genes offers new avenues for cancer treatment, and various targets (e.g., CLK8 [290], mTOR [291]) and drugs (e.g., MLN4924 [292]) are under development with ongoing clinical trials.

Overall, circadian rhythms play a vital role in the development and functioning of immune cells. Current research has confirmed that various immune cell types are regulated by their own circadian rhythm genes as well as environmental factors during development. The distribution and functional status of these cells are also influenced by the expression of circadian rhythm-related products in the microenvironment. For the immune system as a whole, circadian rhythm acts like a control panel, leading to different responses to external stimuli (such as bacterial or viral infections, vaccination, etc.) at different times of the day. For example, receiving the COVID-19 vaccine in the morning may be more beneficial for patients’ health [293]. In recent years, the relationship between energy metabolism and circadian rhythm has also been explored. Hormones closely linked to energy metabolism, such as insulin and glucocorticoids, exhibit circadian rhythm characteristics. These hormones significantly impact immune system function, suggesting that the connection between the immune system and circadian rhythm is not simply a direct regulation. In addition, the liver, as the metabolic hub of the human body, demonstrates distinct circadian rhythm patterns in glucose and lipid metabolism [294]. Another intriguing observation is the loss of control of circadian rhythm-related genes in cancer, which has been demonstrated to have varying effects across different types of cancer [295]. Considering the unique cell cycle regulation and metabolic properties of tumor cells, as well as the intricate relationship among the immune system, energy metabolism, and circadian rhythm, we hypothesize that tumor cells actively modify their rhythmic characteristics to adapt to their specific metabolism and proliferation requirements. By inducing dysregulated rhythms through downstream molecular changes, they can manipulate relevant immune responses to evade attacks from the immune system. Therefore, restoring the circadian rhythm in tumor cells while protecting the immune system from the detrimental effects of circadian rhythm disruptions could potentially serve as a novel therapeutic strategy. Furthermore, monitoring circadian rhythm-related molecules may prove to be an effective approach for cancer prediction and immune function evaluation.

## Data Availability

Data sharing is not applicable to this article as no datasets were generated or analyzed during the current study. All data generated or analyzed during this study are included in this published article or available from the corresponding author on reasonable request.

## References

[1] Scheiermann C, Gibbs J, Ince L, Loudon A (2018). Clocking in to immunity. Nat Rev Immunol.

[2] Zhou L, Zhang Z, Nice E, Huang C, Zhang W, Tang Y (2022). Circadian rhythms and cancers: the intrinsic links and therapeutic potentials. J Hematol Oncol.

[3] Patke A, Young MW, Axelrod S (2020). Molecular mechanisms and physiological importance of circadian rhythms. Nat Rev Mol Cell Biol.

[4] Zhang R, Lahens NF, Ballance HI, Hughes ME, Hogenesch JB (2014). A circadian gene expression atlas in mammals: implications for biology and medicine. Proc Natl Acad Sci USA.

[5] Young MW, Kay SA (2001). Time zones: a comparative genetics of circadian clocks. Nat Rev Genet.

[6] Fulgham CV, Dreyer AP, Nasseri A, Miller AN, Love J, Martin MM (2021). Central and peripheral clock control of circadian feeding rhythms. . Biol Rhythms.

[7] Aton SJ, Herzog ED (2005). Come together, right…now: synchronization of rhythms in a mammalian circadian clock. Neuron.

[8] Schibler U, Gotic I, Saini C, Gos P, Curie T, Emmenegger Y (2015). Clock-Talk: Interactions between Central and Peripheral Circadian Oscillators in Mammals. Cold Spring Harb Symp Quant Biol.

[9] Welsh DK, Yoo SH, Liu AC, Takahashi JS, Kay SA (2004). Bioluminescence imaging of individual fibroblasts reveals persistent, independently phased circadian rhythms of clock gene expression. Curr Biol.

[10] Cajochen C (2007). Alerting effects of light. Sleep Med Rev.

[11] K Pavlova M, Latreille V (2019). Sleep disorders. Am J Med.

[12] Ito E, Inoue Y (2015). The international classification of sleep disorders, third edition. American Academy of Sleep Medicine. Includes bibliographies and index. Nihon Rinsho.

[13] Sun SY, Chen GH (2022). Treatment of circadian rhythm sleep-wake disorders. Curr Neuropharmacol.

[14] Nagtegaal JE, Laurant MW, Kerkhof GA, Smits MG, van der Meer YG, Coenen AM (2000). Effects of melatonin on the quality of life in patients with delayed sleep phase syndrome. J Psychosom Res.

[15] Yagita K, Tamanini F, Yasuda M, Hoeijmakers JH, van der Horst GT, Okamura H (2002). Nucleocytoplasmic shuttling and mCRY-dependent inhibition of ubiquitylation of the mPER2 clock protein. EMBO J.

[16] Guilding C, Scott F, Bechtold DA, Brown TM, Wegner S, Piggins HD (2013). Suppressed cellular oscillations in after-hours mutant mice are associated with enhanced circadian phase-resetting. J Physiol.

[17] Meng QJ, Logunova L, Maywood ES, Gallego M, Lebiecki J, Brown TM (2008). Setting clock speed in mammals: the CK1 epsilon tau mutation in mice accelerates circadian pacemakers by selectively destabilizing PERIOD proteins. Neuron.

[18] Eide EJ, Woolf MF, Kang H, Woolf P, Hurst W, Camacho F (2005). Control of mammalian circadian rhythm by CKIepsilon-regulated proteasome-mediated PER2 degradation. Mol Cell Biol.

[19] Lowrey PL, Shimomura K, Antoch MP, Yamazaki S, Zemenides PD, Ralph MR (2000). Positional syntenic cloning and functional characterization of the mammalian circadian mutation tau. Science.

[20] Maier B, Wendt S, Vanselow JT, Wallach T, Reischl S, Oehmke S (2009). A large-scale functional RNAi screen reveals a role for CK2 in the mammalian circadian clock. Genes Dev.

[21] Toh KL, Jones CR, He Y, Eide EJ, Hinz WA, Virshup DM (2001). An hPer2 phosphorylation site mutation in familial advanced sleep phase syndrome. Science.

[22] Tsuchiya Y, Akashi M, Matsuda M, Goto K, Miyata Y, Node K (2009). Involvement of the protein kinase CK2 in the regulation of mammalian circadian rhythms. Sci Signal.

[23] Vanselow K, Vanselow JT, Westermark PO, Reischl S, Maier B, Korte T (2006). Differential effects of PER2 phosphorylation: molecular basis for the human familial advanced sleep phase syndrome (FASPS). Genes Dev.

[24] Xu Y, Toh KL, Jones CR, Shin JY, Fu YH, Ptácek LJ (2007). Modeling of a human circadian mutation yields insights into clock regulation by PER2. Cell.

[25] Zhou M, Kim JK, Eng GW, Forger DB, Virshup DM (2015). A Period2 phosphoswitch regulates and temperature compensates circadian period. Mol Cell.

[26] Ohsaki K, Oishi K, Kozono Y, Nakayama K, Nakayama KI, Ishida N (2008). The role of {beta}-TrCP1 and {beta}-TrCP2 in circadian rhythm generation by mediating degradation of clock protein PER2. J Biochem.

[27] Shirogane T, Jin J, Ang XL, Harper JW (2005). SCFbeta-TRCP controls clock-dependent transcription via casein kinase 1-dependent degradation of the mammalian period-1 (Per1) protein. J Biol Chem.

[28] Narasimamurthy R, Hunt SR, Lu Y, Fustin JM, Okamura H, Partch CL (2018). CK1δ/ε protein kinase primes the PER2 circadian phosphoswitch. Proc Natl Acad Sci USA.

[29] Gao P, Yoo SH, Lee KJ, Rosensweig C, Takahashi JS, Chen BP (2013). Phosphorylation of the cryptochrome 1 C-terminal tail regulates circadian period length. J Biol Chem.

[30] Hirano A, Shi G, Jones CR, Lipzen A, Pennacchio LA, Xu Y (2016). A Cryptochrome 2 mutation yields advanced sleep phase in humans. Elife.

[31] Hirota T, Lee JW, St John PC, Sawa M, Iwaisako K, Noguchi T (2012). Identification of small molecule activators of cryptochrome. Science.

[32] Khan SK, Xu H, Ukai-Tadenuma M, Burton B, Wang Y, Ueda HR (2012). Identification of a novel cryptochrome differentiating domain required for feedback repression in circadian clock function. J Biol Chem.

[33] Ode KL, Ukai H, Susaki EA, Narumi R, Matsumoto K, Hara J (2017). Knockout-rescue embryonic stem cell-derived mouse reveals circadian-period control by quality and quantity of CRY1. Mol Cell.

[34] Oshima T, Yamanaka I, Kumar A, Yamaguchi J, Nishiwaki-Ohkawa T, Muto K (2015). C-H activation generates period-shortening molecules that target cryptochrome in the mammalian circadian clock. Angew Chem Int Ed Engl.

[35] Patke A, Murphy PJ, Onat OE, Krieger AC, Özçelik T, Campbell SS (2017). Mutation of the human circadian clock gene CRY1 in familial delayed sleep phase disorder. Cell.

[36] Hirano A, Braas D, Fu YH, Ptáček LJ (2017). FAD regulates CRYPTOCHROME protein stability and circadian clock in mice. Cell Rep.

[37] Busino L, Bassermann F, Maiolica A, Lee C, Nolan PM, Godinho SI (2007). SCFFbxl3 controls the oscillation of the circadian clock by directing the degradation of cryptochrome proteins. Science.

[38] Godinho SI, Maywood ES, Shaw L, Tucci V, Barnard AR, Busino L (2007). The after-hours mutant reveals a role for Fbxl3 in determining mammalian circadian period. Science.

[39] Hirano A, Yumimoto K, Tsunematsu R, Matsumoto M, Oyama M, Kozuka-Hata H (2013). FBXL21 regulates oscillation of the circadian clock through ubiquitination and stabilization of cryptochromes. Cell.

[40] Saran AR, Kalinowska D, Oh S, Janknecht R, DiTacchio L (2018). JMJD5 links CRY1 function and proteasomal degradation. PLoS Biol.

[41] Siepka SM, Yoo SH, Park J, Song W, Kumar V, Hu Y (2007). Circadian mutant overtime reveals F-box protein FBXL3 regulation of cryptochrome and period gene expression. Cell.

[42] Yoo SH, Mohawk JA, Siepka SM, Shan Y, Huh SK, Hong HK (2013). Competing E3 ubiquitin ligases govern circadian periodicity by degradation of CRY in nucleus and cytoplasm. Cell.

[43] Zhang Y, Fang B, Emmett MJ, Damle M, Sun Z, Feng D (2015). Discrete functions of nuclear receptor Rev-erbα couple metabolism to the clock. Science.

[44] Cho H, Zhao X, Hatori M, Yu RT, Barish GD, Lam MT (2012). Regulation of circadian behaviour and metabolism by REV-ERB-α and REV-ERB-β. Nature.

[45] Chung S, Lee EJ, Yun S, Choe HK, Park SB, Son HJ (2014). Impact of circadian nuclear receptor REV-ERBα on midbrain dopamine production and mood regulation. Cell.

[46] Takahashi JS (2017). Transcriptional architecture of the mammalian circadian clock. Nat Rev Genet.

[47] Lowrey PL, Takahashi JS (2004). Mammalian circadian biology: elucidating genome-wide levels of temporal organization. Annu Rev Genomics Hum Genet.

[48] Preitner N, Damiola F, Lopez-Molina L, Zakany J, Duboule D, Albrecht U (2002). The orphan nuclear receptor REV-ERBalpha controls circadian transcription within the positive limb of the mammalian circadian oscillator. Cell.

[49] Shearman LP, Sriram S, Weaver DR, Maywood ES, Chaves I, Zheng B (2000). Interacting molecular loops in the mammalian circadian clock. Science.

[50] Farshadi E, van der Horst G, Chaves I (2020). Molecular links between the circadian clock and the cell cycle. J Mol Biol.

[51] He B, Nohara K, Park N, Park YS, Guillory B, Zhao Z (2016). The small molecule nobiletin targets the molecular oscillator to enhance circadian rhythms and protect against metabolic syndrome. Cell Metab.

[52] Shinozaki A, Misawa K, Ikeda Y, Haraguchi A, Kamagata M, Tahara Y (2017). Potent effects of flavonoid nobiletin on amplitude, period, and phase of the circadian clock rhythm in PER2::LUCIFERASE mouse embryonic fibroblasts. PLoS ONE.

[53] Onozuka H, Nakajima A, Matsuzaki K, Shin RW, Ogino K, Saigusa D (2008). Nobiletin, a citrus flavonoid, improves memory impairment and Abeta pathology in a transgenic mouse model of Alzheimer’s disease. J Pharm Exp Ther.

[54] Yabuki Y, Ohizumi Y, Yokosuka A, Mimaki Y, Fukunaga K (2014). Nobiletin treatment improves motor and cognitive deficits seen in MPTP-induced Parkinson model mice. Neuroscience.

[55] Yi LT, Xu HL, Feng J, Zhan X, Zhou LP, Cui CC (2011). Involvement of monoaminergic systems in the antidepressant-like effect of nobiletin. Physiol Behav.

[56] Hanahan D, Weinberg RA (2011). Hallmarks of cancer: the next generation. Cell.

[57] Hanahan D, Weinberg RA (2000). The hallmarks of cancer. Cell.

[58] Vidya MK, Kumar VG, Sejian V, Bagath M, Krishnan G, Bhatta R (2018). Toll-like receptors: significance, ligands, signaling pathways, and functions in mammals. Int Rev Immunol.

[59] Halberg F, johnson EA, brown BW, bittner JJ (1960). Susceptibility rhythm to E. coli endotoxin and bioassay. Proc Soc Exp Biol Med.

[60] Silver AC, Buckley SM, Hughes ME, Hastings AK, Nitabach MN, Fikrig E (2018). Daily oscillations in expression and responsiveness of Toll-like receptors in splenic immune cells. Heliyon.

[61] Kelly B, O’Neill LA (2015). Metabolic reprogramming in macrophages and dendritic cells in innate immunity. Cell Res.

[62] Huang SC, Smith AM, Everts B, Colonna M, Pearce EL, Schilling JD (2016). Metabolic reprogramming mediated by the mTORC2-IRF4 signaling axis is essential for macrophage alternative activation. Immunity.

[63] Timmons GA, O’Siorain JR, Kennedy OD, Curtis AM, Early JO (2020). Innate rhythms: clocks at the center of monocyte and macrophage function. Front Immunol.

[64] Xu H, Li H, Woo SL, Kim SM, Shende VR, Neuendorff N (2014). Myeloid cell-specific disruption of Period1 and Period2 exacerbates diet-induced inflammation and insulin resistance. J Biol Chem.

[65] Liu Z, Gan L, Zhang T, Ren Q, Sun C. Melatonin alleviates adipose inflammation through elevating α-ketoglutarate and diverting adipose-derived exosomes to macrophages in mice. J Pineal Res. 2018;64. 10.1111/jpi.12455 29149454

[66] Ding S, Lin N, Sheng X, Zhao Y, Su Y, Xu L (2019). Melatonin stabilizes rupture-prone vulnerable plaques via regulating macrophage polarization in a nuclear circadian receptor RORα-dependent manner. J Pineal Res.

[67] Allen NC, Philip NH, Hui L, Zhou X, Franklin RA, Kong Y (2019). Desynchronization of the molecular clock contributes to the heterogeneity of the inflammatory response. Sci Signal.

[68] Pariollaud M, Gibbs JE, Hopwood TW, Brown S, Begley N, Vonslow R (2018). Circadian clock component REV-ERBα controls homeostatic regulation of pulmonary inflammation. J Clin Invest.

[69] Gibbs JE, Blaikley J, Beesley S, Matthews L, Simpson KD, Boyce SH (2012). The nuclear receptor REV-ERBα mediates circadian regulation of innate immunity through selective regulation of inflammatory cytokines. Proc Natl Acad Sci USA.

[70] Griffin P, Dimitry JM, Sheehan PW, Lananna BV, Guo C, Robinette ML (2019). Circadian clock protein Rev-erbα regulates neuroinflammation. Proc Natl Acad Sci USA.

[71] Wang S, Lin Y, Yuan X, Li F, Guo L, Wu B (2018). REV-ERBα integrates colon clock with experimental colitis through regulation of NF-κB/NLRP3 axis. Nat Commun.

[72] Pourcet B, Zecchin M, Ferri L, Beauchamp J, Sitaula S, Billon C (2018). Nuclear receptor subfamily 1 group D member 1 regulates circadian activity of NLRP3 inflammasome to reduce the severity of fulminant hepatitis in mice. Gastroenterology.

[73] Wang T, Wang Z, Yang P, Xia L, Zhou M, Wang S (2016). PER1 prevents excessive innate immune response during endotoxin-induced liver injury through regulation of macrophage recruitment in mice. Cell Death Dis.

[74] Silver AC, Arjona A, Walker WE, Fikrig E (2012). The circadian clock controls toll-like receptor 9-mediated innate and adaptive immunity. Immunity.

[75] Narasimamurthy R, Hatori M, Nayak SK, Liu F, Panda S, Verma IM (2012). Circadian clock protein cryptochrome regulates the expression of proinflammatory cytokines. Proc Natl Acad Sci USA.

[76] Yu D, Fang X, Xu Y, Xiao H, Huang T, Zhang Y (2019). Rev-erbα can regulate the NF-κB/NALP3 pathway to modulate lipopolysaccharide-induced acute lung injury and inflammation. Int Immunopharmacology.

[77] McRae HM, Hargreaves DC (2022). Old macrophages lose their (circadian) rhythm. Trends Immunol.

[78] Amir M, Campbell S, Kamenecka TM, Solt LA (2020). Pharmacological modulation and genetic deletion of REV-ERBα and REV-ERBβ regulates dendritic cell development. Biochem Biophys Res Commun.

[79] Nobis CC, Dubeau Laramée G, Kervezee L, Maurice De Sousa D, Labrecque N, Cermakian N (2019). The circadian clock of CD8 T cells modulates their early response to vaccination and the rhythmicity of related signaling pathways. Proc Natl Acad Sci USA.

[80] Holtkamp SJ, Ince LM, Barnoud C, Schmitt MT, Sinturel F, Pilorz V (2021). Circadian clocks guide dendritic cells into skin lymphatics. Nat Immunol.

[81] Wang C, Barnoud C, Cenerenti M, Sun M, Caffa I, Kizil B (2023). Dendritic cells direct circadian anti-tumour immune responses. Nature.

[82] Sun Y, Yang Z, Niu Z, Peng J, Li Q, Xiong W (2006). MOP3, a component of the molecular clock, regulates the development of B cells. Immunology.

[83] Hemmers S, Rudensky AY (2015). The Cell-Intrinsic Circadian Clock Is Dispensable for Lymphocyte Differentiation and Function. Cell Rep.

[84] Druzd D, Matveeva O, Ince L, Harrison U, He W, Schmal C (2017). Lymphocyte Circadian Clocks Control Lymph Node Trafficking and Adaptive Immune Responses. Immunity.

[85] Suzuki K, Hayano Y, Nakai A, Furuta F, Noda M (2016). Adrenergic control of the adaptive immune response by diurnal lymphocyte recirculation through lymph nodes. J Exp Med.

[86] Shimba A, Cui G, Tani-Ichi S, Ogawa M, Abe S, Okazaki F (2018). Glucocorticoids drive diurnal oscillations in T cell distribution and responses by inducing interleukin-7 receptor and CXCR4. Immunity.

[87] Förster R, Davalos-Misslitz AC, Rot A (2008). CCR7 and its ligands: balancing immunity and tolerance. Nat Rev Immunol.

[88] Stein JV, Nombela-Arrieta C (2005). Chemokine control of lymphocyte trafficking: a general overview. Immunology.

[89] Cyster JG, Schwab SR (2012). Sphingosine-1-phosphate and lymphocyte egress from lymphoid organs. Annu Rev Immunol.

[90] Wang Q, Li L, Li C, Cao H, Chen Y, Zhou W (2022). Circadian protein CLOCK modulates regulatory B cell functions of nurses engaging day-night shift rotation. Cell Signal.

[91] Cermakian N, Labrecque N (2023). Regulation of cytotoxic CD8+ T cells by the circadian clock. J Immunol.

[92] Besedovsky L, Born J, Lange T (2014). Endogenous glucocorticoid receptor signaling drives rhythmic changes in human T-cell subset numbers and the expression of the chemokine receptor CXCR4. FASEB J.

[93] Dimitrov S, Benedict C, Heutling D, Westermann J, Born J, Lange T (2009). Cortisol and epinephrine control opposing circadian rhythms in T cell subsets. Blood.

[94] Deprés-Brummer P, Bourin P, Pages N, Metzger G, Lévi F (1997). Persistent T lymphocyte rhythms despite suppressed circadian clock outputs in rats. Am J Physiol.

[95] Kawate T, Abo T, Hinuma S, Kumagai K (1981). Studies of the bioperiodicity of the immune response. II. Co-variations of murine T and B cells and a role of corticosteroid. J Immunol.

[96] Fortier EE, Rooney J, Dardente H, Hardy MP, Labrecque N, Cermakian N (2011). Circadian variation of the response of T cells to antigen. J Immunol.

[97] Wu Y, Tao B, Zhang T, Fan Y, Mao R (2019). Pan-cancer analysis reveals disrupted circadian clock associates with T cell exhaustion. Front Immunol.

[98] Capelle CM, Chen A, Zeng N, Baron A, Grzyb K, Arns T (2022). Stress hormone signalling inhibits Th1 polarization in a CD4 T-cell-intrinsic manner via mTORC1 and the circadian gene PER1. Immunology.

[99] Yang G, Yang Y, Tang H, Yang K (2020). Loss of the clock gene Per1 promotes oral squamous cell carcinoma progression via the AKT/mTOR pathway. Cancer Sci.

[100] Juszczak M, Głabiński A (2009). Th17 cells in the pathogenesis of multiple sclerosis. Postepy Hig Med Dosw.

[101] Yu X, Rollins D, Ruhn KA, Stubblefield JJ, Green CB, Kashiwada M (2013). TH17 cell differentiation is regulated by the circadian clock. Science.

[102] Chang JL, Qiu J (2022). Regulation of ILC3/Th17-mediated intestinal immune response by circadian rhythm. Sichuan Da Xue Xue Bao Yi Xue Ban..

[103] Cheng FL, An YF, Xue JM, Wang YJ, Ding XW, Zhang YT (2022). Circadian rhythm disruption exacerbates Th2-like immune response in murine allergic airway inflammation. Int Forum Allergy Rhinol.

[104] Mandal A, Viswanathan C (2015). Natural killer cells: in health and disease. Hematol Oncol Stem Cell Ther.

[105] Vivier E, Tomasello E, Baratin M, Walzer T, Ugolini S (2008). Functions of natural killer cells. Nat Immunol.

[106] Mattiola I, Pesant M, Tentorio PF, Molgora M, Marcenaro E, Lugli E (2015). Priming of human resting NK cells by autologous M1 macrophages via the engagement of IL-1β, IFN-β, and IL-15 pathways. J Immunol.

[107] Logan RW, Sarkar DK (2012). Circadian nature of immune function. Mol Cell Endocrinol.

[108] Zeng X, Liang C, Yao J (2020). Chronic shift-lag promotes NK cell ageing and impairs immunosurveillance in mice by decreasing the expression of CD122. J. Cell Mol Med.

[109] Kennedy MK, Glaccum M, Brown SN, Butz EA, Viney JL, Embers M (2000). Reversible defects in natural killer and memory CD8 T cell lineages in interleukin 15-deficient mice. J Exp Med.

[110] Chung JW, Yoon SR, Choi I (2008). The regulation of NK cell function and development. Front Biosci.

[111] Benca RM, Quintas J (1997). Sleep and host defenses: a review. Sleep.

[112] Besedovsky L, Lange T, Born J (2012). Sleep and immune function. Pflug Arch.

[113] Lange T, Perras B, Fehm HL, Born J (2003). Sleep enhances the human antibody response to hepatitis A vaccination. Psychosom Med.

[114] Spiegel K, Sheridan JF (2002). Van Cauter E. Effect of sleep deprivation on response to immunization. JAMA.

[115] Dimitrov S, Lange T, Tieken S, Fehm HL, Born J (2004). Sleep associated regulation of T helper 1/T helper 2 cytokine balance in humans. Brain Behav Immun.

[116] Petrovsky N, Harrison LC (1997). Diurnal rhythmicity of human cytokine production: a dynamic disequilibrium in T helper cell type 1/T helper cell type 2 balance. J Immunol.

[117] Petrovsky N, Harrison LC (1998). The chronobiology of human cytokine production. Int Rev Immunol.

[118] Born J, Fehm HL (1998). Hypothalamus-pituitary-adrenal activity during human sleep: a coordinating role for the limbic hippocampal system. Exp Clin Endocrinol Diabetes.

[119] Sadamatsu M, Kato N, Iida H, Takahashi S, Sakaue K, Takahashi K (1995). The 24-hour rhythms in plasma growth hormone, prolactin and thyroid stimulating hormone: effect of sleep deprivation. J Neuroendocrinol.

[120] Spiegel K, Luthringer R, Follenius M, Schaltenbrand N, Macher JP, Muzet A (1995). Temporal relationship between prolactin secretion and slow-wave electroencephalic activity during sleep. Sleep.

[121] Alexander J, Satoskar AR, Russell DG (1999). Leishmania species: models of intracellular parasitism. J Cell Sci.

[122] Karupiah G (1998). Type 1 and type 2 cytokines in antiviral defense. Vet Immunol Immunopathol.

[123] Kühlwein E, Irwin M (2001). Melatonin modulation of lymphocyte proliferation and Th1/Th2 cytokine expression. J Neuroimmunol.

[124] Lio D, Balistreri CR, Candore G, D’Anna C, Di Lorenzo G, Gervasi F (2000). In vitro treatment with interleukin-2 normalizes type-1 cytokine production by lymphocytes from elderly. Immunopharmacol Immunotoxicol.

[125] Van Cauter E, Leproult R, Plat L (2000). Age-related changes in slow wave sleep and REM sleep and relationship with growth hormone and cortisol levels in healthy men. JAMA.

[126] Höflich C, Döcke WD, Busch A, Kern F, Volk HD (1998). CD45RA(bright)/CD11a(bright) CD8+ T cells: effector T cells. Int Immunol.

[127] von Andrian UH, Mackay CR (2000). T-cell function and migration. Two sides of the same coin. N Engl J Med.

[128] Irwin MR, Wang M, Campomayor CO, Collado-Hidalgo A, Cole S (2006). Sleep deprivation and activation of morning levels of cellular and genomic markers of inflammation. Arch Intern Med.

[129] Phillips B, Mannino DM (2005). Does insomnia kill. Sleep.

[130] Vgontzas AN, Zoumakis E, Bixler EO, Lin HM, Follett H, Kales A (2004). Adverse effects of modest sleep restriction on sleepiness, performance, and inflammatory cytokines. J Clin Endocrinol Metab.

[131] Shearer WT, Reuben JM, Mullington JM, Price NJ, Lee BN, Smith EO (2001). Soluble TNF-alpha receptor 1 and IL-6 plasma levels in humans subjected to the sleep deprivation model of spaceflight. J Allergy Clin Immunol.

[132] Meier-Ewert HK, Ridker PM, Rifai N, Regan MM, Price NJ, Dinges DF (2004). Effect of sleep loss on C-reactive protein, an inflammatory marker of cardiovascular risk. J Am Coll Cardiol.

[133] Godinho-Silva C, Domingues RG, Rendas M, Raposo B, Ribeiro H, da Silva JA (2019). Light-entrained and brain-tuned circadian circuits regulate ILC3s and gut homeostasis. Nature.

[134] Stokes K, Cooke A, Chang H, Weaver DR, Breault DT, Karpowicz P (2017). The circadian clock gene BMAL1 coordinates intestinal regeneration. Cell Mol Gastroenterol Hepatol.

[135] Qin B, Deng Y (2015). Overexpression of circadian clock protein cryptochrome (CRY) 1 alleviates sleep deprivation-induced vascular inflammation in a mouse model. Immunol Lett.

[136] Magzal F, Shochat T, Haimov I, Tamir S, Asraf K, Tuchner-Arieli M (2022). Increased physical activity improves gut microbiota composition and reduces short-chain fatty acid concentrations in older adults with insomnia. Sci Rep.

[137] Thaiss CA, Zeevi D, Levy M, Zilberman-Schapira G, Suez J, Tengeler AC (2014). Transkingdom control of microbiota diurnal oscillations promotes metabolic homeostasis. Cell.

[138] Yang DF, Huang WC, Wu CW, Huang CY, Yang Y, Tung YT (2023). Acute sleep deprivation exacerbates systemic inflammation and psychiatry disorders through gut microbiota dysbiosis and disruption of circadian rhythms. Microbiol Res.

[139] Koritala B, Porter KI, Arshad OA, Gajula RP, Mitchell HD, Arman T (2021). Night shift schedule causes circadian dysregulation of DNA repair genes and elevated DNA damage in humans. J Pineal Res.

[140] Haus EL, Smolensky MH (2013). Shift work and cancer risk: potential mechanistic roles of circadian disruption, light at night, and sleep deprivation. Sleep Med Rev.

[141] James SM, Honn KA, Gaddameedhi S, Van Dongen H (2017). Shift work: disrupted circadian rhythms and sleep-implications for health and well-being. Curr Sleep Med Rep.

[142] Savvidis C, Koutsilieris M (2012). Circadian rhythm disruption in cancer biology. Mol Med.

[143] Sahar S, Sassone-Corsi P (2009). Metabolism and cancer: the circadian clock connection. Nat Rev Cancer.

[144] Sancar A, Lindsey-Boltz LA, Gaddameedhi S, Selby CP, Ye R, Chiou YY (2015). Circadian clock, cancer, and chemotherapy. Biochemistry.

[145] Yang Y, Liu Z, Selby CP, Sancar A (2019). Long-term, genome-wide kinetic analysis of the effect of the circadian clock and transcription on the repair of cisplatin-DNA adducts in the mouse liver. J Biol Chem.

[146] Gaucher J, Montellier E, Sassone-Corsi P (2018). Molecular cogs: interplay between circadian clock and cell cycle. Trends Cell Biol.

[147] Miller BH, McDearmon EL, Panda S, Hayes KR, Zhang J, Andrews JL (2007). Circadian and CLOCK-controlled regulation of the mouse transcriptome and cell proliferation. Proc Natl Acad Sci USA.

[148] Scheiermann C, Kunisaki Y, Frenette PS (2013). Circadian control of the immune system. Nat Rev Immunol.

[149] Keller M, Mazuch J, Abraham U, Eom GD, Herzog ED, Volk HD (2009). A circadian clock in macrophages controls inflammatory immune responses. Proc Natl Acad Sci USA.

[150] Masri S, Cervantes M, Sassone-Corsi P (2013). The circadian clock and cell cycle: interconnected biological circuits. Curr Opin Cell Biol.

[151] Roenneberg T, Merrow M (2016). The circadian clock and human health. Curr Biol.

[152] Depner CM, Melanson EL, McHill AW, Wright KP (2018). Mistimed food intake and sleep alters 24-hour time-of-day patterns of the human plasma proteome. Proc Natl Acad Sci USA.

[153] Kervezee L, Cuesta M, Cermakian N, Boivin DB (2018). Simulated night shift work induces circadian misalignment of the human peripheral blood mononuclear cell transcriptome. Proc Natl Acad Sci USA.

[154] Skene DJ, Skornyakov E, Chowdhury NR, Gajula RP, Middleton B, Satterfield BC (2018). Separation of circadian- and behavior-driven metabolite rhythms in humans provides a window on peripheral oscillators and metabolism. Proc Natl Acad Sci USA.

[155] Resuehr D, Wu G, Johnson RL, Young ME, Hogenesch JB, Gamble KL (2019). Shift work disrupts circadian regulation of the transcriptome in hospital nurses. J Biol Rhythms.

[156] Humans, IMotIoCHt, Work iNS. Night shift work. 2020. Lyon (FR). other.

[157] Papagiannakopoulos T, Bauer MR, Davidson SM, Heimann M, Subbaraj L, Bhutkar A (2016). Circadian rhythm disruption promotes lung tumorigenesis. Cell Metab.

[158] Lee Y, Lahens NF, Zhang S, Bedont J, Field JM, Sehgal A (2019). G1/S cell cycle regulators mediate effects of circadian dysregulation on tumor growth and provide targets for timed anticancer treatment. PLoS Biol.

[159] Van Dycke KC, Rodenburg W, van Oostrom CT, van Kerkhof LW, Pennings JL, Roenneberg T (2015). Chronically alternating light cycles increase breast cancer risk in mice. Curr Biol.

[160] Aiello I, Fedele M, Román F, Marpegan L, Caldart C, Chiesa JJ (2020). Circadian disruption promotes tumor-immune microenvironment remodeling favoring tumor cell proliferation. Sci Adv.

[161] Bentivoglio M, Grassi-Zucconi G (1997). The pioneering experimental studies on sleep deprivation. Sleep.

[162] Rechtschaffen A, Gilliland MA, Bergmann BM, Winter JB (1983). Physiological correlates of prolonged sleep deprivation in rats. Science.

[163] Shaw PJ, Tononi G, Greenspan RJ, Robinson DF (2002). Stress response genes protect against lethal effects of sleep deprivation in Drosophila. Nature.

[164] Stephenson R, Chu KM, Lee J. Prolonged deprivation of sleep-like rest raises metabolic rate in the Pacific beetle cockroach, Diploptera punctata (Eschscholtz). J Exp Biol. 2007;210:2540–7. 10.1242/jeb.005322 17601958

[165] Donlea JM. Roles for sleep in memory: insights from the fly. Curr Opin Neurobiol. 2019;54:120–6. 10.1016/j.conb.2018.10.006 PMC6361691 30366270

[166] Killgore WD. Effects of sleep deprivation on cognition. Prog Brain Res. 2010;185:105–29. 10.1016/B978-0-444-53702-7.00007-5 21075236

[167] Krause AJ, Simon EB, Mander BA, Greer SM, Saletin JM, Goldstein-Piekarski AN (2017). The sleep-deprived human brain. Nat Rev Neurosci.

[168] Reimund E. The free radical flux theory of sleep. Med Hypotheses. 1994;43:231–3. 10.1016/0306-9877(94)90071-x 7838006

[169] Alzoubi KH, Khabour OF, Rashid BA, Damaj IM, Salah HA (2012). The neuroprotective effect of vitamin E on chronic sleep deprivation-induced memory impairment: the role of oxidative stress. Behav Brain Res.

[170] Hill VM, O’Connor RM, Sissoko GB, Irobunda IS, Leong S, Canman JC (2018). A bidirectional relationship between sleep and oxidative stress in Drosophila. PLoS Biol.

[171] Kanazawa L, Vecchia DD, Wendler EM, Hocayen P, Dos Reis Lívero FA, Stipp MC (2016). Quercetin reduces manic-like behavior and brain oxidative stress induced by paradoxical sleep deprivation in mice. Free Radic Biol Med.

[172] Süer C, Dolu N, Artis AS, Sahin L, Yilmaz A, Cetin A (2011). The effects of long-term sleep deprivation on the long-term potentiation in the dentate gyrus and brain oxidation status in rats. Neurosci Res.

[173] Villafuerte G, Miguel-Puga A, Rodríguez EM, Machado S, Manjarrez E, Arias-Carrión O (2015). Sleep deprivation and oxidative stress in animal models: a systematic review. Oxid Med Cell Longev.

[174] Vaccaro A, Kaplan Dor Y, Nambara K, Pollina EA, Lin C, Greenberg ME (2020). Sleep loss can cause death through accumulation of reactive oxygen species in the gut. Cell.

[175] Balaban RS, Nemoto S, Finkel T (2005). Mitochondria, oxidants, and aging. Cell.

[176] Shimizu Y, Hendershot LM (2009). Oxidative folding: cellular strategies for dealing with the resultant equimolar production of reactive oxygen species. Antioxid Redox Signal.

[177] Cao SS, Kaufman RJ (2014). Endoplasmic reticulum stress and oxidative stress in cell fate decision and human disease. Antioxid Redox Signal.

[178] Santos CX, Tanaka LY, Wosniak J, Laurindo FR (2009). Mechanisms and implications of reactive oxygen species generation during the unfolded protein response: roles of endoplasmic reticulum oxidoreductases, mitochondrial electron transport, and NADPH oxidase. Antioxid Redox Signal.

[179] Zeeshan HM, Lee GH, Kim HR, Chae HJ (2016). Endoplasmic Reticulum Stress and Associated ROS. Int J Mol Sci.

[180] Cirelli C, Faraguna U, Tononi G (2006). Changes in brain gene expression after long-term sleep deprivation. J Neurochem.

[181] Eiland MM, Ramanathan L, Gulyani S, Gilliland M, Bergmann BM, Rechtschaffen A (2002). Increases in amino-cupric-silver staining of the supraoptic nucleus after sleep deprivation. Brain Res.

[182] Gopalakrishnan A, Ji LL, Cirelli C (2004). Sleep deprivation and cellular responses to oxidative stress. Sleep.

[183] Hipólide DC, D’Almeida V, Raymond R, Tufik S, Nobrega JN (2002). Sleep deprivation does not affect indices of necrosis or apoptosis in rat brain. Int J Neurosci.

[184] Brown MK, Strus E, Naidoo N (2017). Reduced sleep during social isolation leads to cellular stress and induction of the unfolded protein response. Sleep.

[185] Nikonova EV, Naidoo N, Zhang L, Romer M, Cater JR, Scharf MT (2010). Changes in components of energy regulation in mouse cortex with increases in wakefulness. Sleep.

[186] Jones S, Pfister-Genskow M, Benca RM, Cirelli C (2008). Molecular correlates of sleep and wakefulness in the brain of the white-crowned sparrow. J Neurochem.

[187] Cirelli C, LaVaute TM, Tononi G (2005). Sleep and wakefulness modulate gene expression in Drosophila. J Neurochem.

[188] Cirelli C (2006). Cellular consequences of sleep deprivation in the brain. Sleep Med Rev.

[189] Ali T, Choe J, Awab A, Wagener TL, Orr WC (2013). Sleep, immunity and inflammation in gastrointestinal disorders. World J Gastroenterol.

[190] Khanijow V, Prakash P, Emsellem HA, Borum ML, Doman DB (2015). Sleep dysfunction and gastrointestinal diseases. Gastroenterol Hepatol.

[191] Parekh PJ, Oldfield EC, Johnson DA (2018). Wake-up call to clinicians: the impact of sleep dysfunction on gastrointestinal health and disease. J Clin Gastroenterol.

[192] Bhattacharyya A, Chattopadhyay R, Mitra S, Crowe SE (2014). Oxidative stress: an essential factor in the pathogenesis of gastrointestinal mucosal diseases. Physiol Rev.

[193] Campbell EL, Colgan SP (2019). Control and dysregulation of redox signalling in the gastrointestinal tract. Nat Rev Gastroenterol Hepatol.

[194] Pérez S, Taléns-Visconti R, Rius-Pérez S, Finamor I, Sastre J (2017). Redox signaling in the gastrointestinal tract. Free Radic Biol Med.

[195] Aviello G, Knaus UG (2017). ROS in gastrointestinal inflammation: Rescue Or Sabotage. Br J Pharm.

[196] Lasry A, Zinger A, Ben-Neriah Y (2016). Inflammatory networks underlying colorectal cancer. Nat Immunol.

[197] Chen GY, Nuñez G (2010). Sterile inflammation: sensing and reacting to damage. Nat Rev Immunol.

[198] Donath MY, Shoelson SE (2011). Type 2 diabetes as an inflammatory disease. Nat Rev Immunol.

[199] Libby P, Lichtman AH, Hansson GK (2013). Immune effector mechanisms implicated in atherosclerosis: from mice to humans. Immunity.

[200] Bruunsgaard H, Ladelund S, Pedersen AN, Schroll M, Jørgensen T, Pedersen BK (2003). Predicting death from tumour necrosis factor-alpha and interleukin-6 in 80-year-old people. Clin Exp Immunol.

[201] Volpato S, Guralnik JM, Ferrucci L, Balfour J, Chaves P, Fried LP (2001). Cardiovascular disease, interleukin-6, and risk of mortality in older women: the women’s health and aging study. Circulation.

[202] Smagula SF, Stone KL, Redline S, Ancoli-Israel S, Barrett-Connor E, Lane NE (2016). Actigraphy- and polysomnography-measured sleep disturbances, inflammation, and mortality among older men. Psychosom Med.

[203] Ridker PM (2016). From C-reactive protein to interleukin-6 to interleukin-1: moving upstream to identify novel targets for atheroprotection. Circ Res.

[204] Grandner MA, Alfonso-Miller P, Fernandez-Mendoza J, Shetty S, Shenoy S, Combs D (2016). Sleep: important considerations for the prevention of cardiovascular disease. Curr Opin Cardiol.

[205] Ferrie JE, Kivimäki M, Akbaraly TN, Singh-Manoux A, Miller MA, Gimeno D (2013). Associations between change in sleep duration and inflammation: findings on C-reactive protein and interleukin 6 in the Whitehall II Study. Am J Epidemiol.

[206] Carroll JE, Irwin MR, Stein Merkin S, Seeman TE (2015). Sleep and multisystem biological risk: a population-based study. PLoS ONE.

[207] Clark AJ, Dich N, Lange T, Jennum P, Hansen AM, Lund R (2014). Impaired sleep and allostatic load: cross-sectional results from the Danish Copenhagen Aging and Midlife Biobank. Sleep Med.

[208] Dowd JB, Goldman N, Weinstein M (2011). Sleep duration, sleep quality, and biomarkers of inflammation in a Taiwanese population. Ann Epidemiol.

[209] Patel SR, Zhu X, Storfer-Isser A, Mehra R, Jenny NS, Tracy R (2009). Sleep duration and biomarkers of inflammation. Sleep.

[210] Prather AA, Vogelzangs N, Penninx BW (2015). Sleep duration, insomnia, and markers of systemic inflammation: results from the Netherlands Study of Depression and Anxiety (NESDA). J Psychiatr Res.

[211] Jackowska M, Steptoe A (2015). Sleep and future cardiovascular risk: prospective analysis from the English Longitudinal Study of Ageing. Sleep Med.

[212] McDade TW, Hawkley LC, Cacioppo JT (2006). Psychosocial and behavioral predictors of inflammation in middle-aged and older adults: the Chicago health, aging, and social relations study. Psychosom Med.

[213] Prather AA, Marsland AL, Hall M, Neumann SA, Muldoon MF, Manuck SB (2009). Normative variation in self-reported sleep quality and sleep debt is associated with stimulated pro-inflammatory cytokine production. Biol Psychol.

[214] Suarez EC (2008). Self-reported symptoms of sleep disturbance and inflammation, coagulation, insulin resistance and psychosocial distress: evidence for gender disparity. Brain Behav Immun.

[215] Taheri S, Austin D, Lin L, Nieto FJ, Young T, Mignot E (2007). Correlates of serum C-reactive protein (CRP)-no association with sleep duration or sleep disordered breathing. Sleep.

[216] Prather AA, Epel ES, Cohen BE, Neylan TC, Whooley MA (2013). Gender differences in the prospective associations of self-reported sleep quality with biomarkers of systemic inflammation and coagulation: findings from the Heart and Soul Study. J Psychiatr Res.

[217] Obayashi K, Saeki K, Kurumatani N (2016). Gender differences in the association between objective sleep quality and leukocyte count: The HEIJO-KYO cohort. Physiol Behav.

[218] Blair LM, Porter K, Leblebicioglu B, Christian LM (2015). Poor sleep quality and associated inflammation predict preterm birth: heightened risk among African Americans. Sleep.

[219] Okun ML, Luther JF, Wisniewski SR, Wisner KL (2013). Disturbed sleep and inflammatory cytokines in depressed and nondepressed pregnant women: an exploratory analysis of pregnancy outcomes. Psychosom Med.

[220] Tsujimura T, Matsuo Y, Keyaki T, Sakurada K, Imanishi J (2009). Correlations of sleep disturbance with the immune system in type 2 diabetes mellitus. Diabetes Res Clin Pr.

[221] Besedovsky L, Lange T, Haack M (2019). The sleep-immune crosstalk in health and disease. Physiol Rev.

[222] Opp MR (2005). Cytokines and sleep. Sleep Med Rev.

[223] Lou X, Wang H, Tu Y, Tan W, Jiang C, Sun J (2021). Alterations of sleep quality and circadian rhythm genes expression in elderly thyroid nodule patients and risks associated with thyroid malignancy. Sci Rep.

[224] Zhou L, Luo Z, Li Z, Huang Q (2020). Circadian clock is associated with tumor microenvironment in kidney renal clear cell carcinoma. Aging.

[225] Ramos CA, Ouyang C, Qi Y, Chung Y, Cheng CT, LaBarge MA (2020). A non-canonical function of BMAL1 metabolically limits obesity-promoted triple-negative breast cancer. iScience.

[226] Chen P, Hsu WH, Chang A, Tan Z, Lan Z, Zhou A (2020). Circadian regulator CLOCK recruits immune-suppressive microglia into the GBM tumor microenvironment. Cancer Discov.

[227] He L, Fan Y, Zhang Y, Tu T, Zhang Q, Yuan F (2022). Single-cell transcriptomic analysis reveals circadian rhythm disruption associated with poor prognosis and drug-resistance in lung adenocarcinoma. J Pineal Res.

[228] Chun SK, Fortin BM, Fellows RC, Habowski AN, Verlande A, Song WA (2022). Disruption of the circadian clock drives Apc loss of heterozygosity to accelerate colorectal cancer. Sci Adv.

[229] Hunt T, Sassone-Corsi P (2007). Riding tandem: circadian clocks and the cell cycle. Cell.

[230] Satyanarayana A, Kaldis P (2009). Mammalian cell-cycle regulation: several Cdks, numerous cyclins and diverse compensatory mechanisms. Oncogene.

[231] Quail DF, Joyce JA (2013). Microenvironmental regulation of tumor progression and metastasis. Nat Med.

[232] Xuan W, Khan F, James CD, Heimberger AB, Lesniak MS, Chen P (2021). Circadian regulation of cancer cell and tumor microenvironment crosstalk. Trends Cell Biol.

[233] Chen DS, Mellman I (2013). Oncology meets immunology: the cancer-immunity cycle. Immunity.

[234] Raskov H, Orhan A, Christensen JP, Gögenur I (2021). Cytotoxic CD8(+) T cells in cancer and cancer immunotherapy. Br J Cancer.

[235] Marvel D, Gabrilovich DI (2015). Myeloid-derived suppressor cells in the tumor microenvironment: expect the unexpected. J Clin Invest.

[236] Roberts NT, MacDonald CR, Mohammadpour H, Antoch MP, Repasky EA (2022). Circadian rhythm disruption increases tumor growth rate and accumulation of myeloid-derived suppressor cells. Adv Biol.

[237] Méndez-Ferrer S, Lucas D, Battista M, Frenette PS (2008). Haematopoietic stem cell release is regulated by circadian oscillations. Nature.

[238] Logan RW, Arjona A, Sarkar DK (2011). Role of sympathetic nervous system in the entrainment of circadian natural-killer cell function. Brain Behav Immun.

[239] Pathria P, Louis TL, Varner JA (2019). Targeting tumor-associated macrophages in cancer. Trends Immunol.

[240] Alexander RK, Liou YH, Knudsen NH, Starost KA, Xu C, Hyde AL (2020). Bmal1 integrates mitochondrial metabolism and macrophage activation. Elife.

[241] Early JO, Menon D, Wyse CA, Cervantes-Silva MP, Zaslona Z, Carroll RG (2018). Circadian clock protein BMAL1 regulates IL-1β in macrophages via NRF2. Proc Natl Acad Sci USA.

[242] Nguyen KD, Fentress SJ, Qiu Y, Yun K, Cox JS, Chawla A (2013). Circadian gene Bmal1 regulates diurnal oscillations of Ly6C(hi) inflammatory monocytes. Science.

[243] Huang C, Zhang C, Cao Y, Li J, Bi F (2023). Major roles of the circadian clock in cancer. Cancer Biol Med.

[244] Sato S, Sakurai T, Ogasawara J, Takahashi M, Izawa T, Imaizumi K (2014). A circadian clock gene, Rev-erbα, modulates the inflammatory function of macrophages through the negative regulation of Ccl2 expression. J Immunol.

[245] Mantovani A, Sica A, Sozzani S, Allavena P, Vecchi A, Locati M (2004). The chemokine system in diverse forms of macrophage activation and polarization. Trends Immunol.

[246] Verreck FA, de Boer T, Langenberg DM, Hoeve MA, Kramer M, Vaisberg E (2004). Human IL-23-producing type 1 macrophages promote but IL-10-producing type 2 macrophages subvert immunity to (myco)bacteria. Proc Natl Acad Sci USA.

[247] Qin C, Zhou LQ, Ma XT, Hu ZW, Yang S, Chen M (2019). Dual functions of microglia in ischemic stroke. Neurosci Bull.

[248] Li X, Guan J, Jiang Z, Cheng S, Hou W, Yao J (2021). Microglial exosome miR-7239-3p promotes glioma progression by regulating circadian genes. Neurosci Bull.

[249] Newman AM, Alizadeh AA (2016). High-throughput genomic profiling of tumor-infiltrating leukocytes. Curr Opin Immunol.

[250] He W, Holtkamp S, Hergenhan SM, Kraus K, de Juan A, Weber J (2018). Circadian expression of migratory factors establishes lineage-specific signatures that guide the homing of leukocyte subsets to tissues. Immunity.

[251] Gao Y, Meng D, Sun N, Zhu Z, Zhao R, Lu C (2014). Clock upregulates intercellular adhesion molecule-1 expression and promotes mononuclear cells adhesion to endothelial cells. Biochem Biophys Res Commun.

[252] Hadadi E, Taylor W, Li XM, Aslan Y, Villote M, Rivière J (2020). Chronic circadian disruption modulates breast cancer stemness and immune microenvironment to drive metastasis in mice. Nat Commun.

[253] Hu X, Liu X, Moisan J, Wang Y, Lesch CA, Spooner C (2016). Synthetic RORγ agonists regulate multiple pathways to enhance antitumor immunity. Oncoimmunology.

[254] Lee IK, Song H, Kim H, Kim IS, Tran NL, Kim SH, et al. RORα regulates cholesterol metabolism of CD8+ T cells for anticancer immunity. Cancers. 2020;12:1733. 10.3390/cancers12071733 PMC7407186 32610705

[255] Kaplon J, van Dam L, Peeper D (2015). Two-way communication between the metabolic and cell cycle machineries: the molecular basis. Cell Cycle.

[256] de Winter L, Schepers LW, Cuaresma M, Barbosa MJ, Martens DE, Wijffels RH (2014). Circadian rhythms in the cell cycle and biomass composition of Neochloris oleoabundans under nitrogen limitation. J Biotechnol.

[257] Krishnaiah SY, Wu G, Altman BJ, Growe J, Rhoades SD, Coldren F (2017). Clock regulation of metabolites reveals coupling between transcription and metabolism. Cell Metab.

[258] Janich P, Pascual G, Merlos-Suárez A, Batlle E, Ripperger J, Albrecht U (2011). The circadian molecular clock creates epidermal stem cell heterogeneity. Nature.

[259] Geyfman M, Kumar V, Liu Q, Ruiz R, Gordon W, Espitia F (2012). Brain and muscle Arnt-like protein-1 (BMAL1) controls circadian cell proliferation and susceptibility to UVB-induced DNA damage in the epidermis. Proc Natl Acad Sci USA.

[260] Plikus MV, Vollmers C, de la Cruz D, Chaix A, Ramos R, Panda S (2013). Local circadian clock gates cell cycle progression of transient amplifying cells during regenerative hair cycling. Proc Natl Acad Sci USA.

[261] Karpowicz P, Zhang Y, Hogenesch JB, Emery P, Perrimon N (2013). The circadian clock gates the intestinal stem cell regenerative state. Cell Rep.

[262] Weger M, Diotel N, Dorsemans AC, Dickmeis T, Weger BD (2017). Stem cells and the circadian clock. Dev Biol.

[263] Kowalska E, Ripperger JA, Hoegger DC, Bruegger P, Buch T, Birchler T (2013). NONO couples the circadian clock to the cell cycle. Proc Natl Acad Sci USA.

[264] Bjarnason GA, Jordan RC, Wood PA, Li Q, Lincoln DW, Sothern RB (2001). Circadian expression of clock genes in human oral mucosa and skin: association with specific cell-cycle phases. Am J Pathol.

[265] Bjarnason GA, Jordan RC, Sothern RB (1999). Circadian variation in the expression of cell-cycle proteins in human oral epithelium. Am J Pathol.

[266] Soták M, Sumová A, Pácha J (2014). Cross-talk between the circadian clock and the cell cycle in cancer. Ann Med.

[267] Matsuo T, Yamaguchi S, Mitsui S, Emi A, Shimoda F, Okamura H (2003). Control mechanism of the circadian clock for timing of cell division in vivo. Science.

[268] Gréchez-Cassiau A, Rayet B, Guillaumond F, Teboul M, Delaunay F (2008). The circadian clock component BMAL1 is a critical regulator of p21WAF1/CIP1 expression and hepatocyte proliferation. J Biol Chem.

[269] Miki T, Matsumoto T, Zhao Z, Lee CC (2013). p53 regulates Period2 expression and the circadian clock. Nat Commun.

[270] Bee L, Marini S, Pontarin G, Ferraro P, Costa R, Albrecht U (2015). Nucleotide excision repair efficiency in quiescent human fibroblasts is modulated by circadian clock. Nucleic Acids Res.

[271] Oklejewicz M, Destici E, Tamanini F, Hut RA, Janssens R, van der Horst GT (2008). Phase resetting of the mammalian circadian clock by DNA damage. Curr Biol.

[272] Masri S, Kinouchi K, Sassone-Corsi P (2015). Circadian clocks, epigenetics, and cancer. Curr Opin Oncol.

[273] Scheiermann C, Kunisaki Y, Lucas D, Chow A, Jang JE, Zhang D (2012). Adrenergic nerves govern circadian leukocyte recruitment to tissues. Immunity.

[274] Wang Y, Sun N, Lu C, Bei Y, Qian R, Hua L (2017). Upregulation of circadian gene ‘hClock’ contribution to metastasis of colorectal cancer. Int J Oncol.

[275] Hu Z, Brooks SA, Dormoy V, Hsu CW, Hsu HY, Lin LT (2015). Assessing the carcinogenic potential of low-dose exposures to chemical mixtures in the environment: focus on the cancer hallmark of tumor angiogenesis. Carcinogenesis.

[276] Jiang X, Wang J, Deng X, Xiong F, Zhang S, Gong Z (2020). The role of microenvironment in tumor angiogenesis. J Exp Clin Cancer Res.

[277] Koyanagi S, Kuramoto Y, Nakagawa H, Aramaki H, Ohdo S, Soeda S (2003). A molecular mechanism regulating circadian expression of vascular endothelial growth factor in tumor cells. Cancer Res.

[278] Zhou J, Li X, Zhang M, Gong J, Li Q, Shan B (2020). The aberrant expression of rhythm genes affects the genome instability and regulates the cancer immunity in pan-cancer. Cancer Med.

[279] Shi SQ, Ansari TS, McGuinness OP, Wasserman DH, Johnson CH (2013). Circadian disruption leads to insulin resistance and obesity. Curr Biol.

[280] Kettner NM, Mayo SA, Hua J, Lee C, Moore DD, Fu L (2015). Circadian dysfunction induces leptin resistance in mice. Cell Metab.

[281] Gabrilovich DI, Nagaraj S (2009). Myeloid-derived suppressor cells as regulators of the immune system. Nat Rev Immunol.

[282] Michaeli J, Shaul ME, Mishalian I, Hovav AH, Levy L, Zolotriov L (2017). Tumor-associated neutrophils induce apoptosis of non-activated CD8 T-cells in a TNFα and NO-dependent mechanism, promoting a tumor-supportive environment. Oncoimmunology.

[283] Feng S, Cheng X, Zhang L, Lu X, Chaudhary S, Teng R (2018). Myeloid-derived suppressor cells inhibit T cell activation through nitrating LCK in mouse cancers. Proc Natl Acad Sci USA.

[284] Peranzoni E, Lemoine J, Vimeux L, Feuillet V, Barrin S, Kantari-Mimoun C (2018). Macrophages impede CD8 T cells from reaching tumor cells and limit the efficacy of anti-PD-1 treatment. Proc Natl Acad Sci USA.

[285] Zhao Y, Liu M, Chan XY, Tan SY, Subramaniam S, Fan Y (2017). Uncovering the mystery of opposite circadian rhythms between mouse and human leukocytes in humanized mice. Blood.

[286] Müller A, Homey B, Soto H, Ge N, Catron D, Buchanan ME (2001). Involvement of chemokine receptors in breast cancer metastasis. Nature.

[287] Helbig G, Christopherson KW, Bhat-Nakshatri P, Kumar S, Kishimoto H, Miller KD (2003). NF-kappaB promotes breast cancer cell migration and metastasis by inducing the expression of the chemokine receptor CXCR4. J Biol Chem.

[288] Nagarsheth N, Wicha MS, Zou W (2017). Chemokines in the cancer microenvironment and their relevance in cancer immunotherapy. Nat Rev Immunol.

[289] Cermakian N, Stegeman SK, Tekade K, Labrecque N (2022). Circadian rhythms in adaptive immunity and vaccination. Semin Immunopathol.

[290] Doruk YU, Yarparvar D, Akyel YK, Gul S, Taskin AC, Yilmaz F (2020). A CLOCK-binding small molecule disrupts the interaction between CLOCK and BMAL1 and enhances circadian rhythm amplitude. J Biol Chem.

[291] Ramanathan C, Kathale ND, Liu D, Lee C, Freeman DA, Hogenesch JB (2018). mTOR signaling regulates central and peripheral circadian clock function. PLoS Genet.

[292] Zhang S, Zhang J, Deng Z, Liu H, Mao W, Jiang F (2016). Circadian clock components RORα and Bmal1 mediate the anti-proliferative effect of MLN4924 in osteosarcoma cells. Oncotarget.

[293] Zhang H, Liu Y, Liu D, Zeng Q, Li L, Zhou Q (2021). Time of day influences immune response to an inactivated vaccine against SARS-CoV-2. Cell Res.

[294] Bass J (2012). Circadian topology of metabolism. Nature.

[295] Masri S, Sassone-Corsi P (2018). The emerging link between cancer, metabolism, and circadian rhythms. Nat Med.

